# Supplementary material for: TDP-1, the Caenorhabditis elegans ortholog of TDP-43, limits the accumulation of double-stranded RNA
Source: EMBO J. 2014 Nov 12;33(24):2947–66. doi: 10.15252/embj.201488740 (PMC4282642; doi:10.15252/embj.201488740)
Supplement: Supplementary file 1 — Supplementary Figures [file embj0033-2947-sd1.pdf]

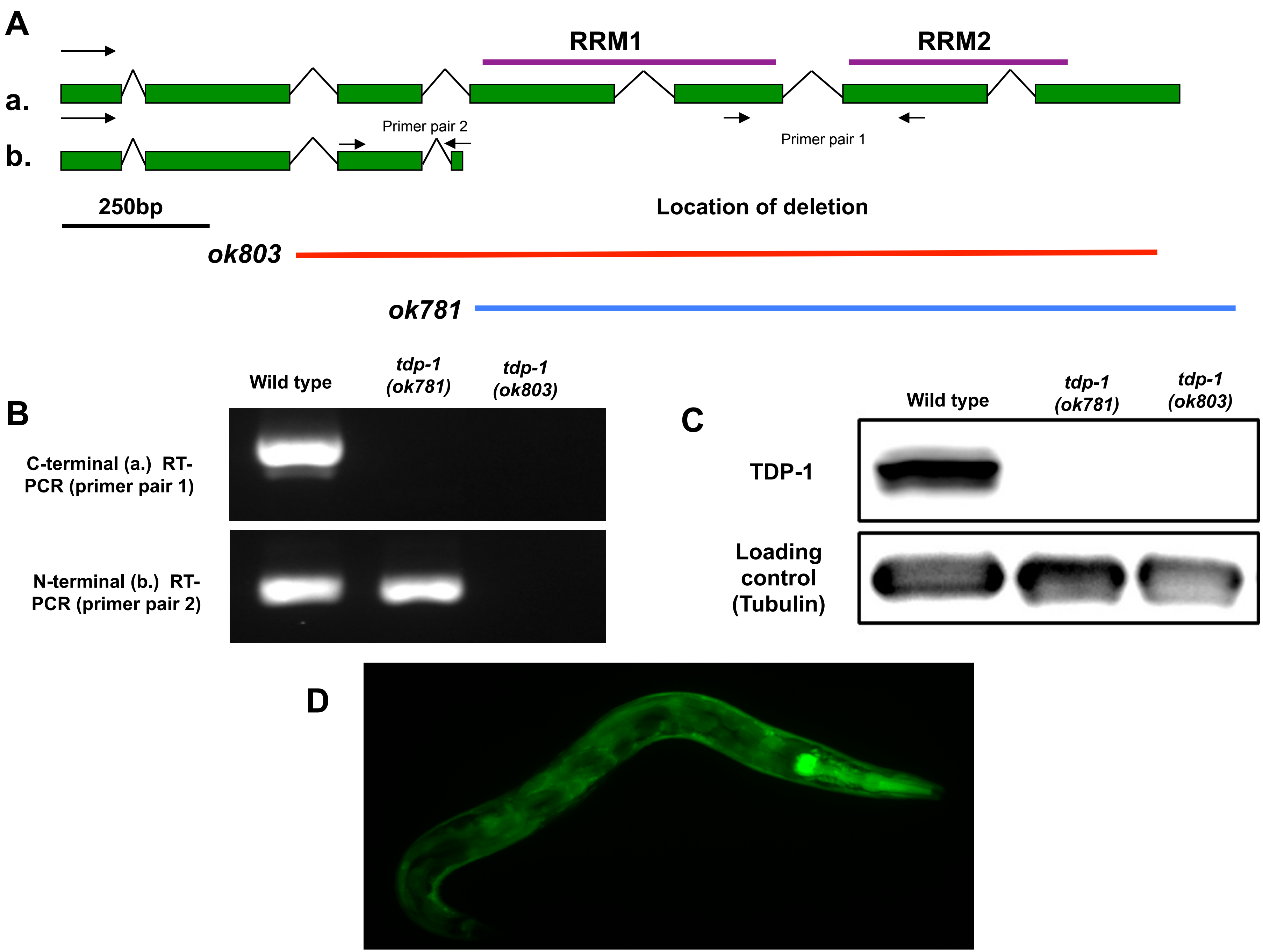
A
RRM1
RRM2
a.
b.
Primer pair 2
Primer pair 1
250bp
Location of deletion
ok803
ok781
B
Wild type
tdp-1 (ok781)
tdp-1 (ok803)
C-terminal (a.) RT-PCR (primer pair 1)
N-terminal (b.) RT-PCR (primer pair 2)
C
Wild type
tdp-1 (ok781)
tdp-1 (ok803)
TDP-1
Loading control (Tubulin)
D

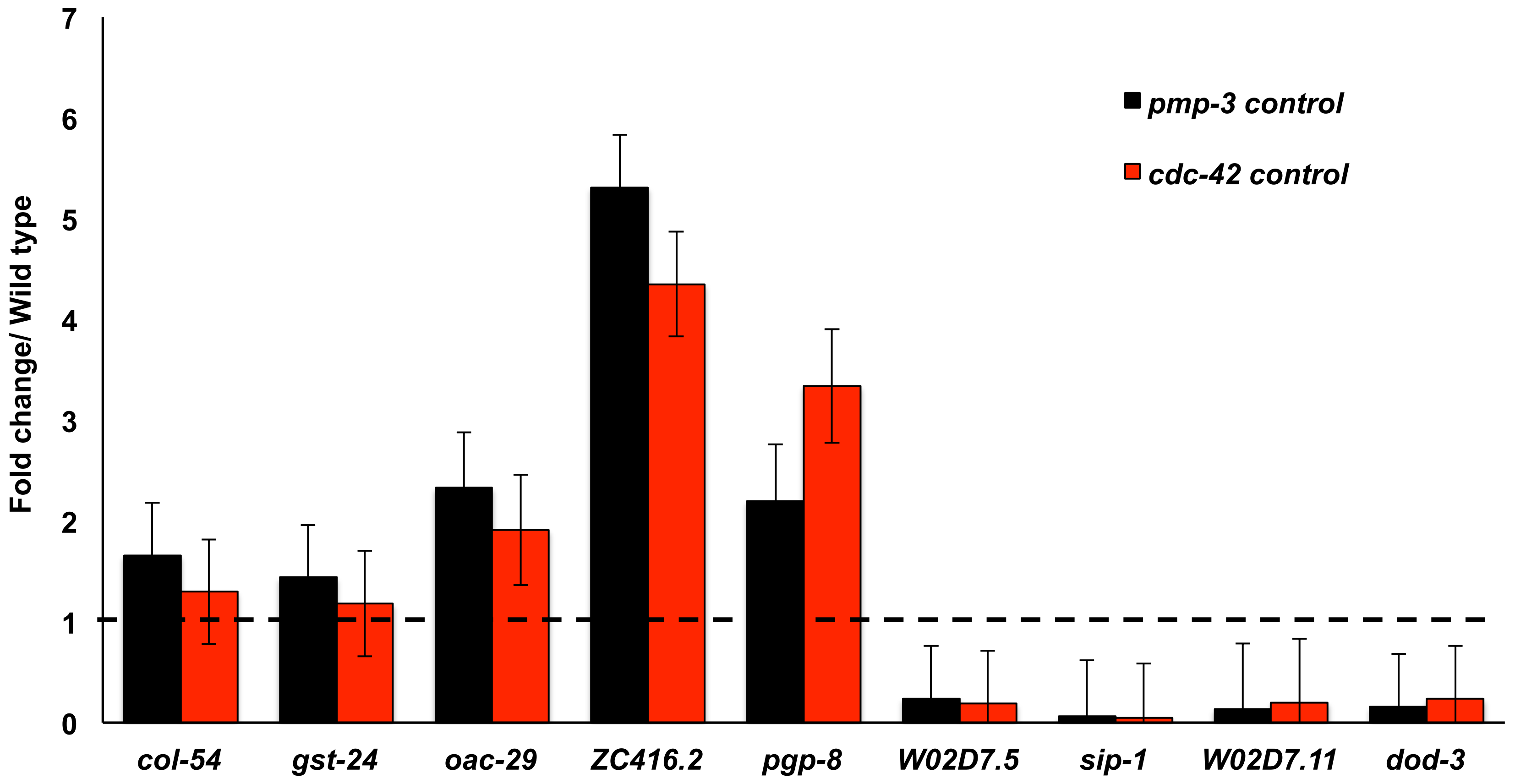

pmp-3 control
cdc-42 control
Fold change/ Wild type
0
1
2
3
4
5
6
7
col-54
gst-24
oac-29
ZC416.2
pgp-8
W02D7.5
sip-1
W02D7.11
dod-3

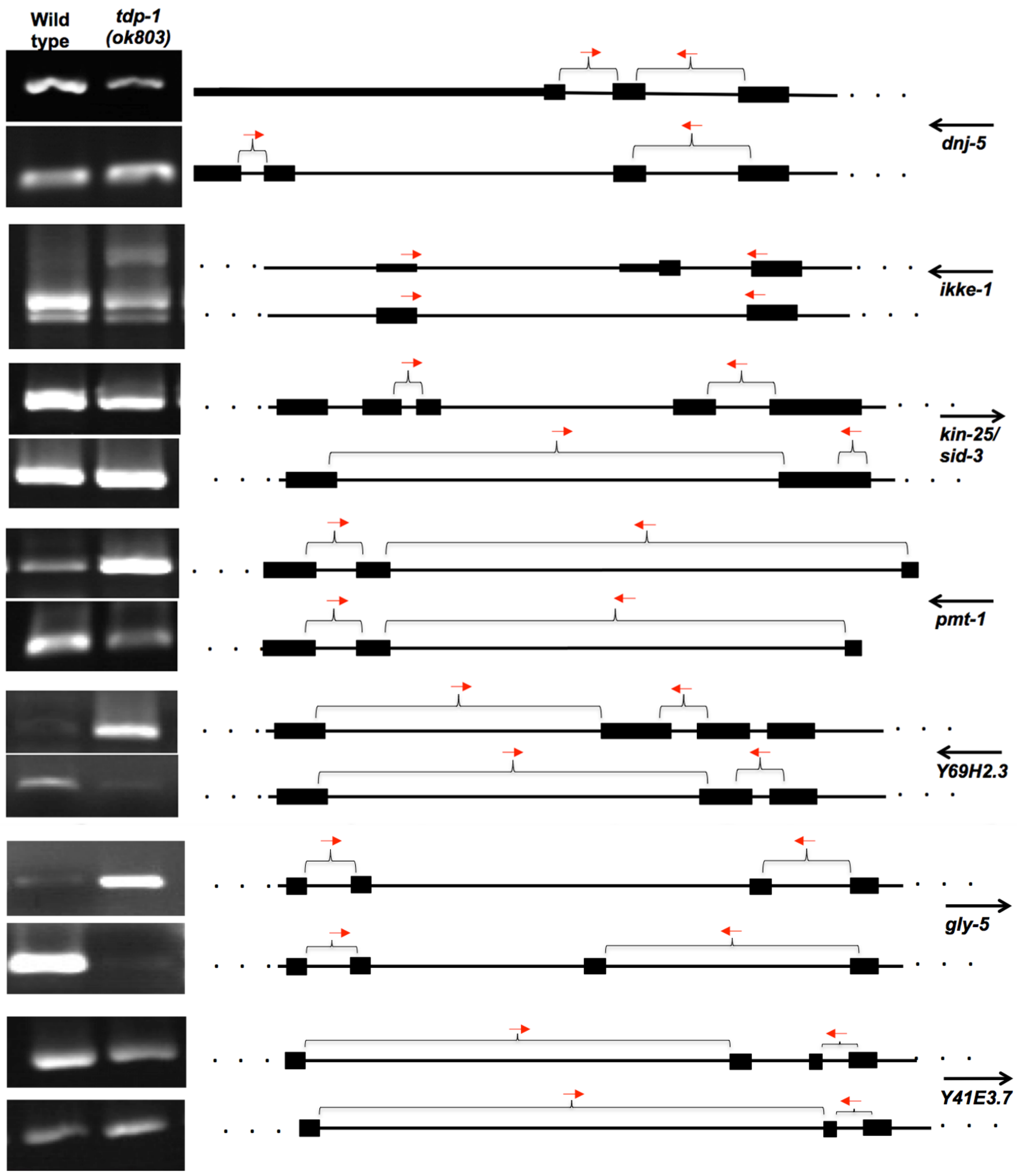
Wild type
tdp-1 (ok803)
dnj-5
ikke-1
kin-25/ sid-3
pmt-1
Y69H2.3
gly-5
Y41E3.7

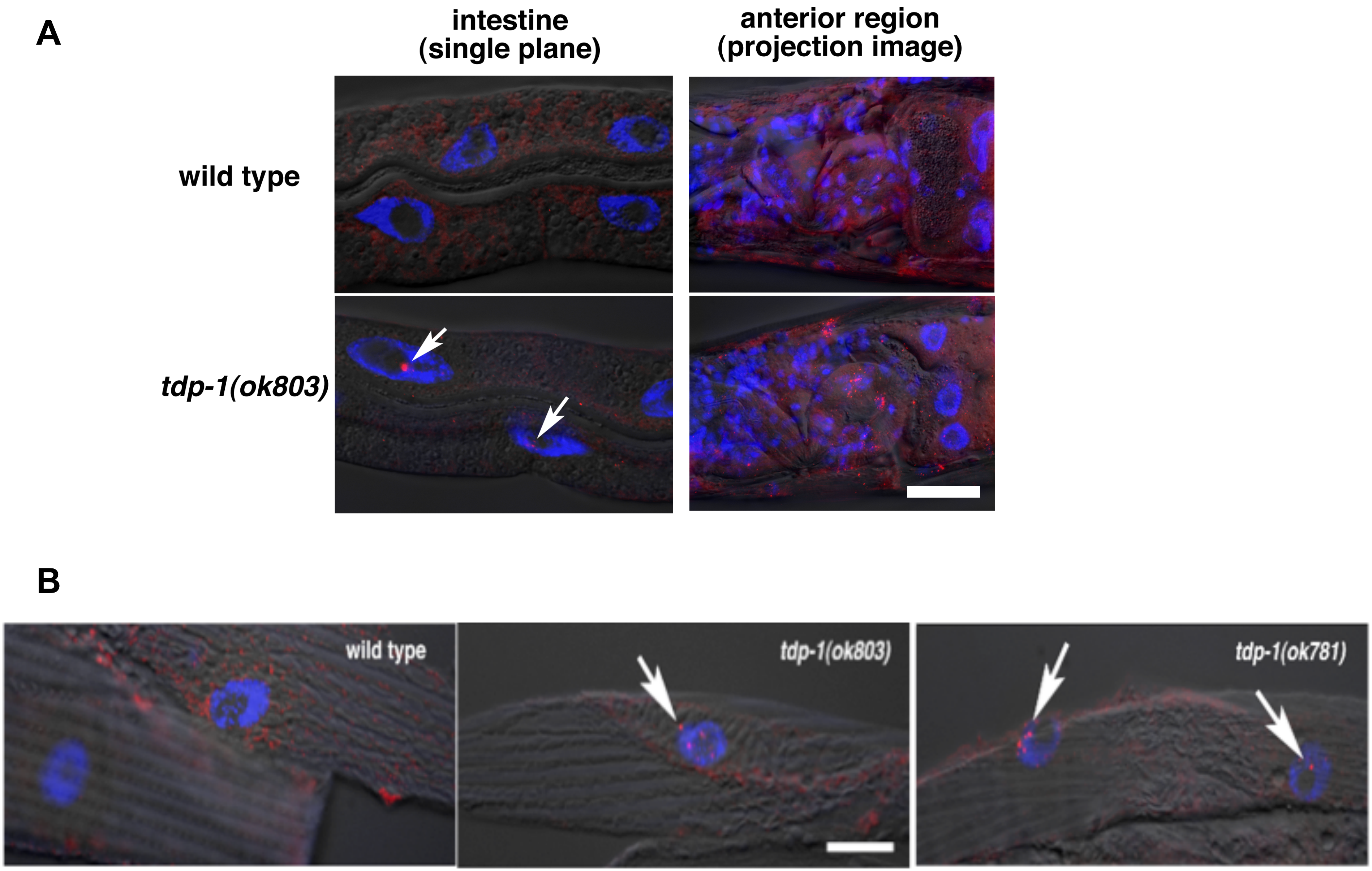

A
intestine (single plane)
anterior region (projection image)
wild type
tdp-1(ok803)
B
wild type
tdp-1(ok803)
tdp-1(ok781)

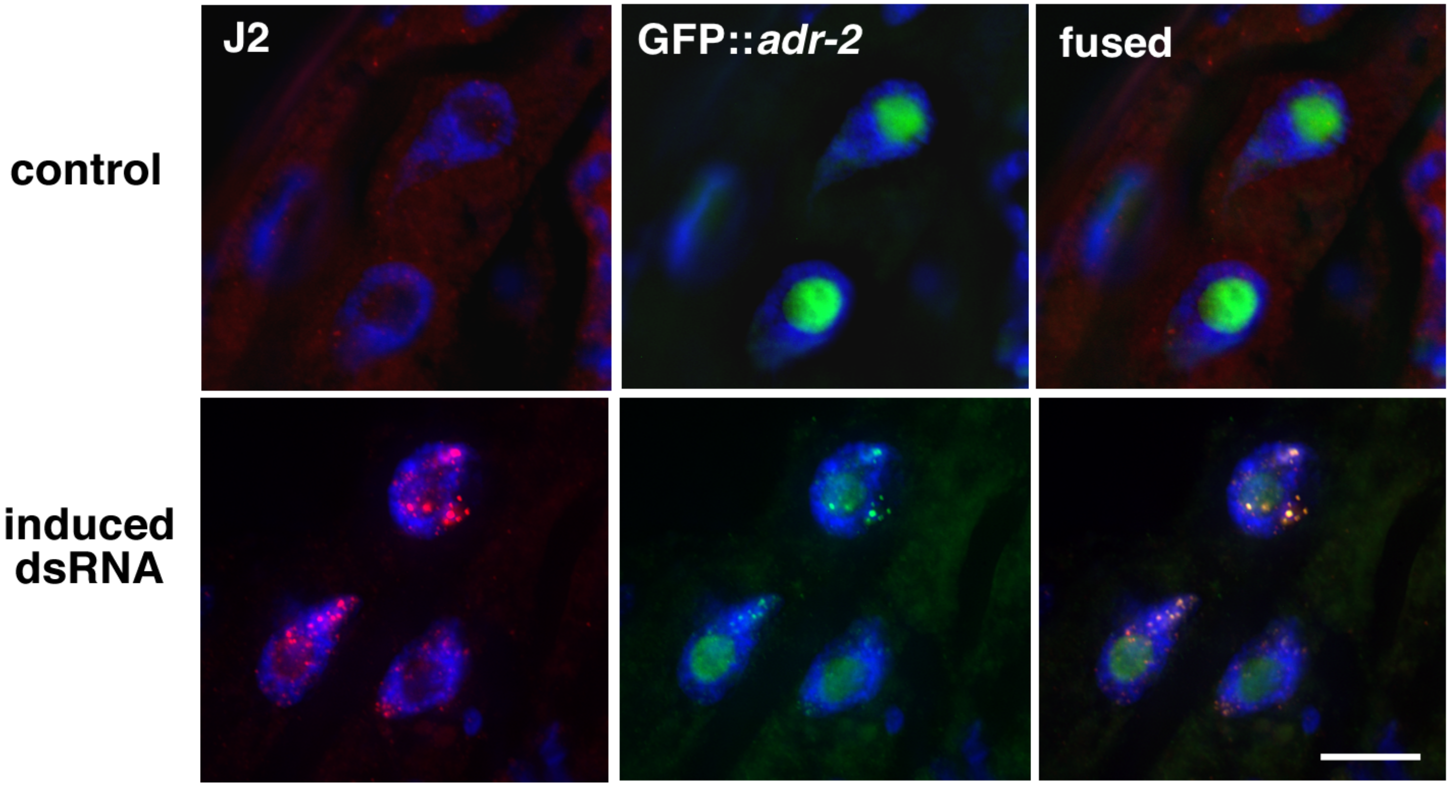

J2
GFP::*adr-2*
fused
control
induced dsRNA

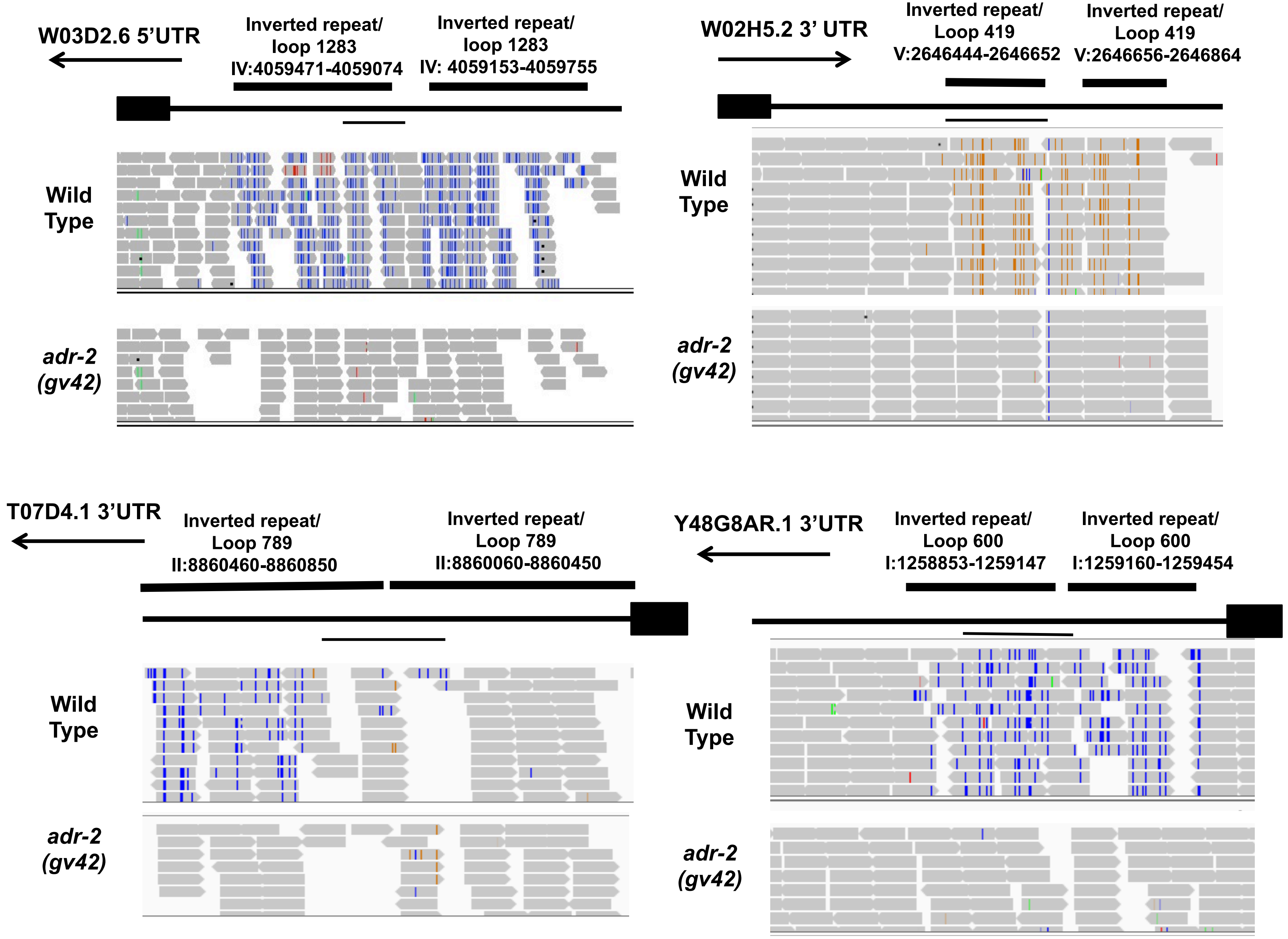

W03D2.6 5'UTR
Inverted repeat/
loop 1283
IV:4059471-4059074
Inverted repeat/
loop 1283
IV: 4059153-4059755
Wild
Type
adr-2
(gv42)
W02H5.2 3' UTR
Inverted repeat/
Loop 419
V:2646444-2646652
Inverted repeat/
Loop 419
V:2646656-2646864
Wild
Type
adr-2
(gv42)
T07D4.1 3'UTR
Inverted repeat/
Loop 789
II:8860460-8860850
Inverted repeat/
Loop 789
II:8860060-8860450
Wild
Type
adr-2
(gv42)
Y48G8AR.1 3'UTR
Inverted repeat/
Loop 600
I:1258853-1259147
Inverted repeat/
Loop 600
I:1259160-1259454
Wild
Type
adr-2
(gv42)

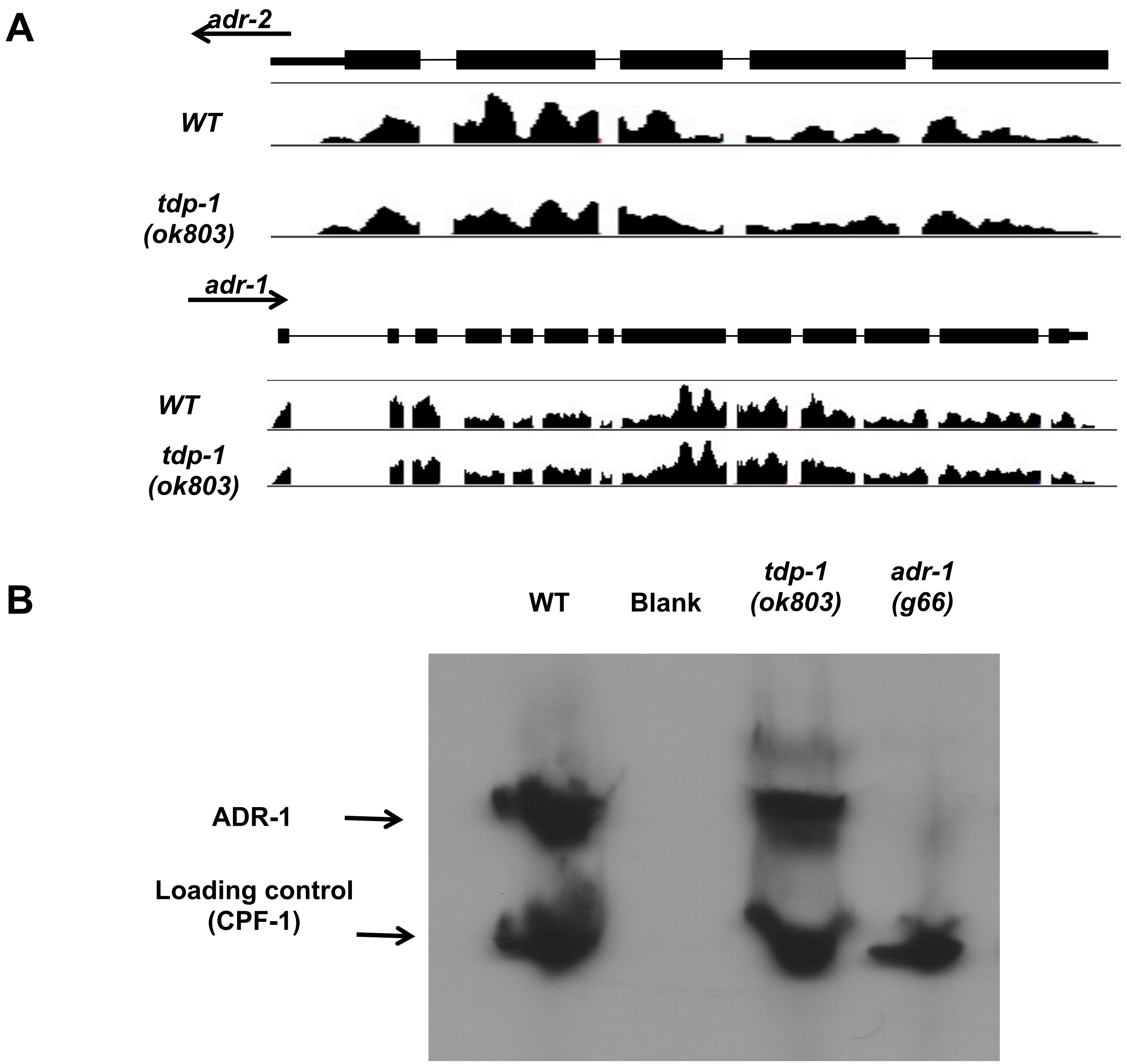

A
adr-2
WT
tdp-1 (ok803)
adr-1
WT
tdp-1 (ok803)
B
WT
Blank
tdp-1 (ok803)
adr-1 (g66)
ADR-1
Loading control (CPF-1)

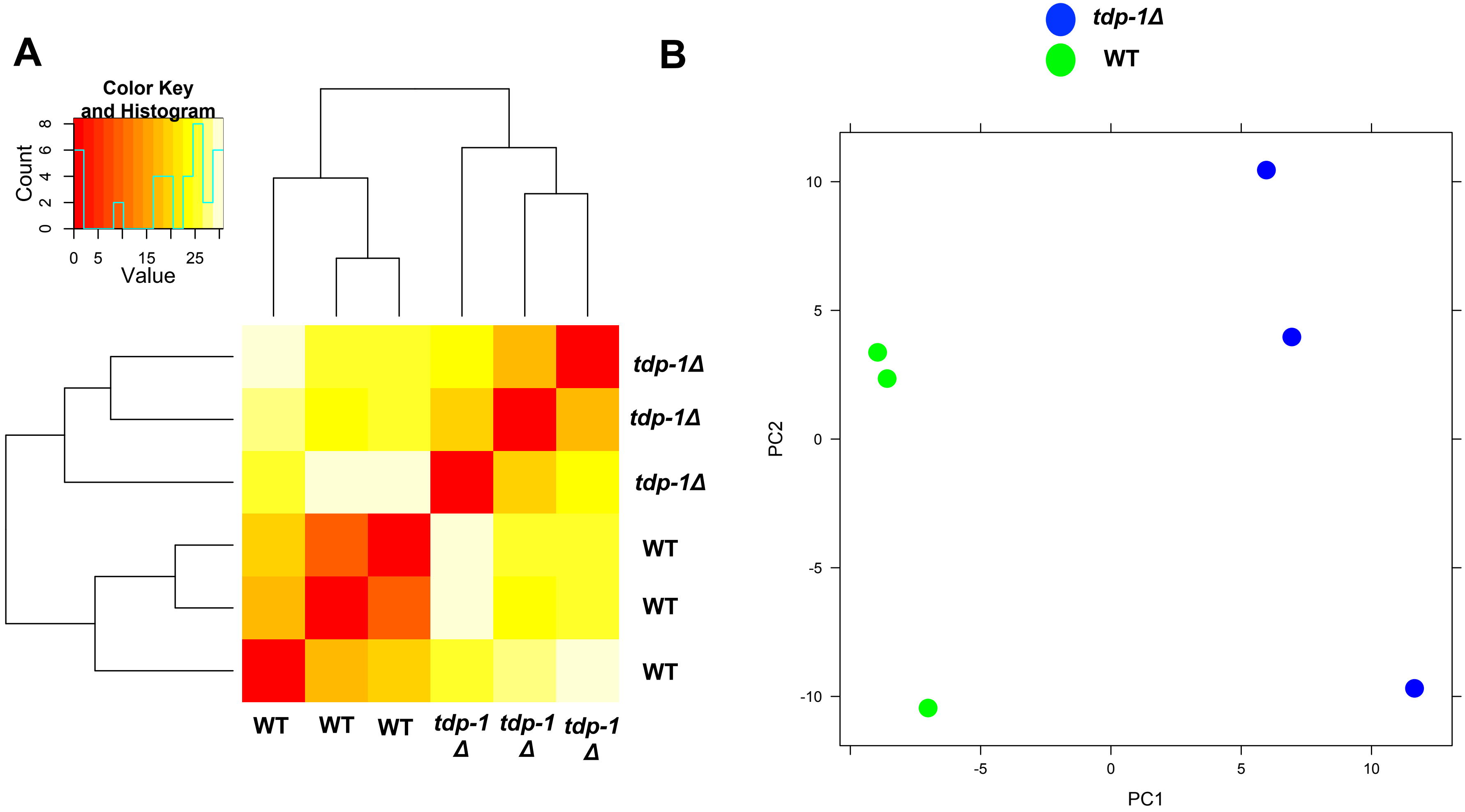
A
Color Key and Histogram
Count
0 2 4 6 8
0 5 15 25
Value
tdp-1Δ
tdp-1Δ
tdp-1Δ
WT
WT
WT
WT WT WT tdp-1 Δ tdp-1 Δ tdp-1 Δ
B
tdp-1Δ
WT
PC2
10 5 0 -5 -10
-5 0 5 10
PC1

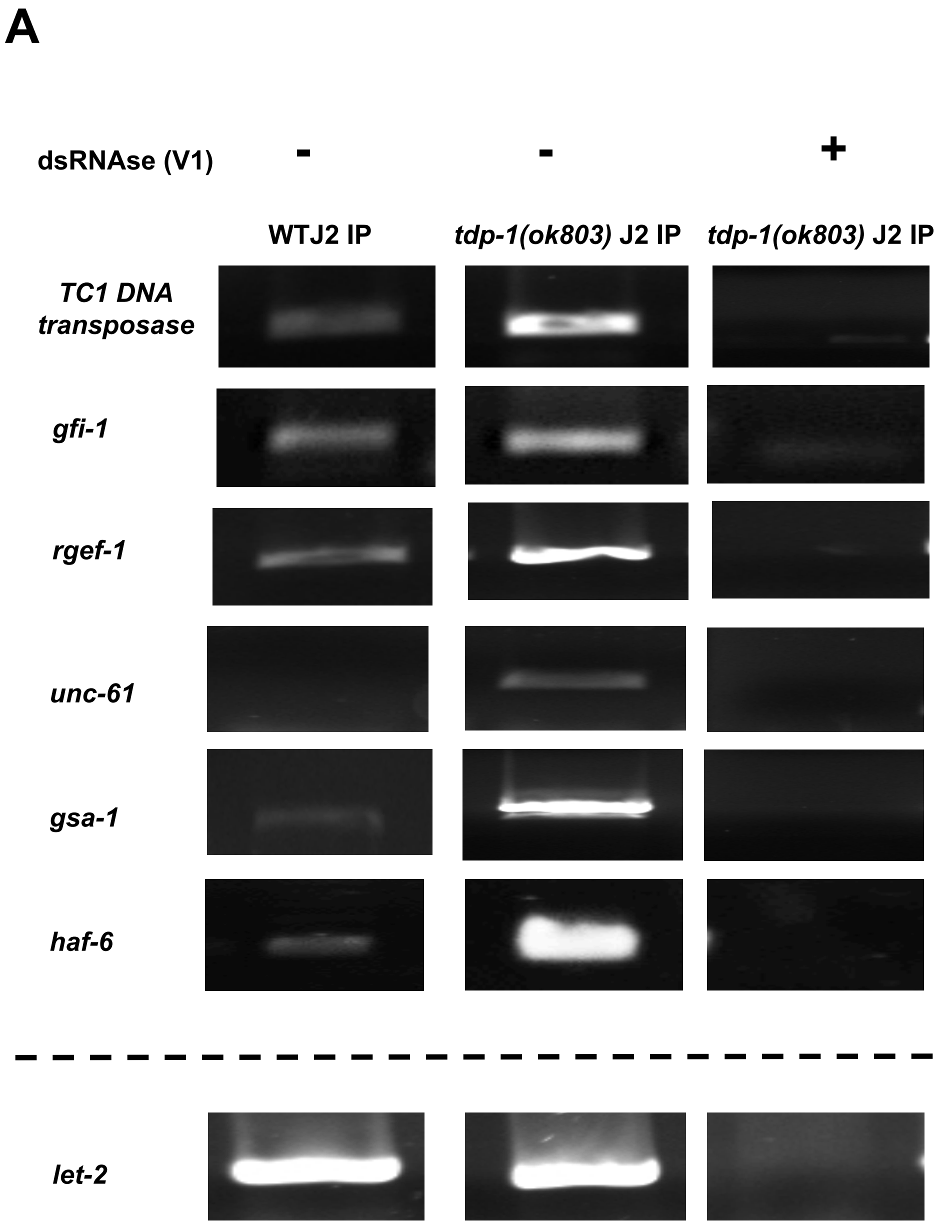

B

Fold increase J2-IP/input

	WTJ2 IP	*tdp-1(ok803)* J2 IP
TC1 DNA transposase	3.3-fold	7-fold
gfi-1	2-fold	3.7-fold
rgef-1	1.1-fold	2.1-fold
unc-61	1.2-fold	1.9-fold
gsa-1	1.3 fold	5-fold
haf-6	2.4-fold	4.6-fold
let-2	1.1-fold	1.2-fold

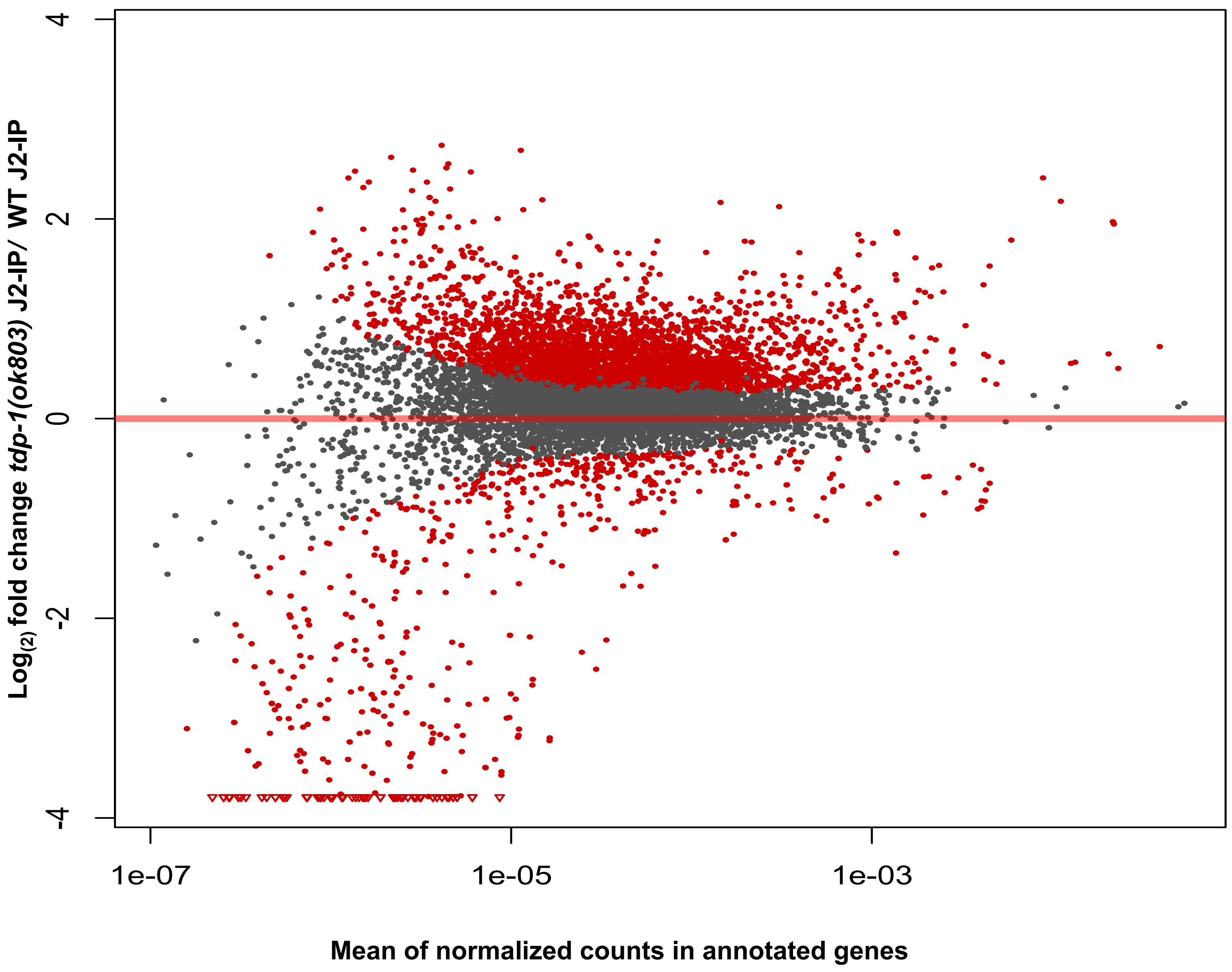

$Log_{(2)}$ fold change *tdp-1(ok803)* J2-IP/ WT J2-IP
4
2
0
-2
-4
1e-07
1e-05
1e-03
Mean of normalized counts in annotated genes

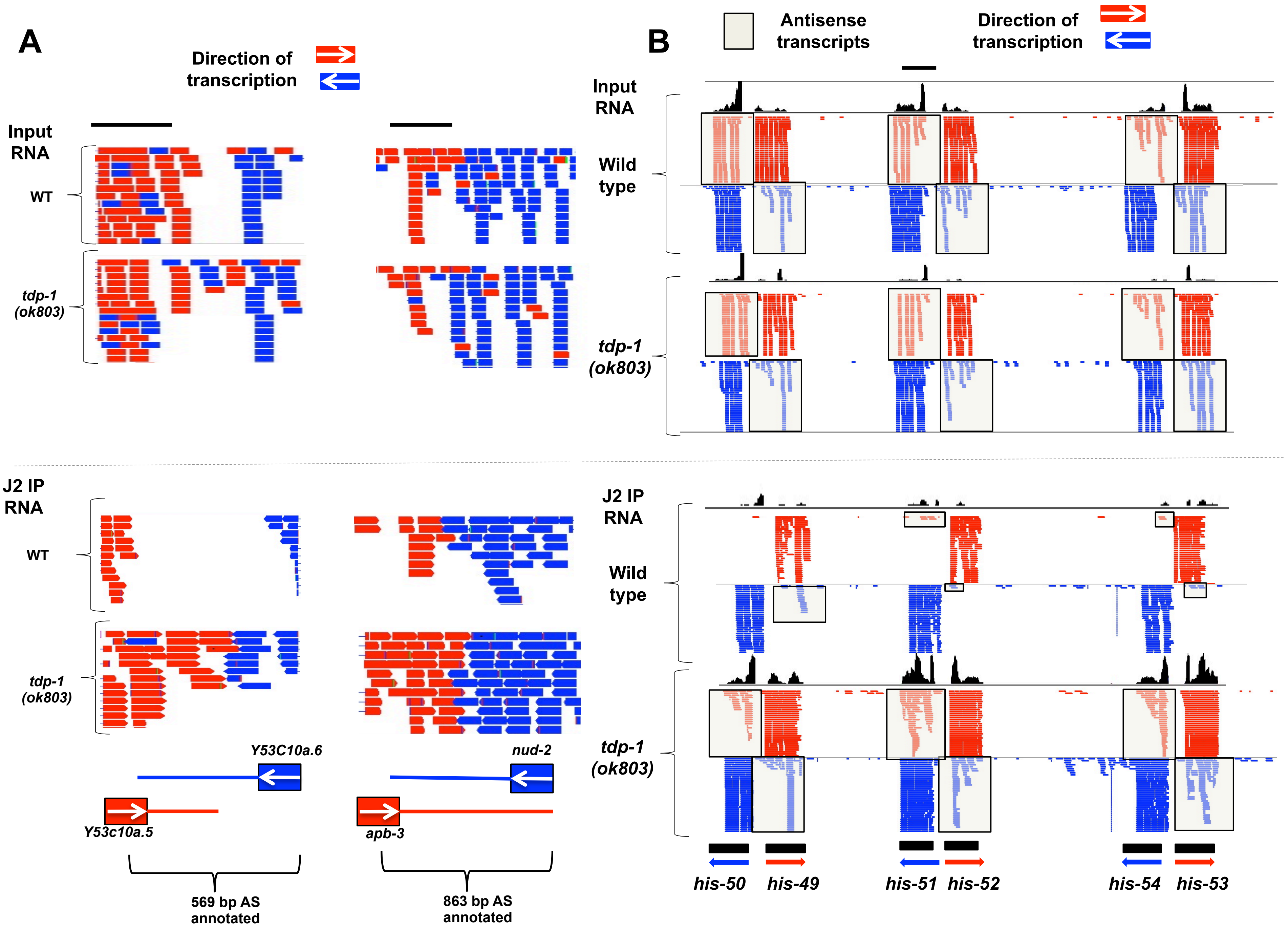

A
Direction of transcription
Input RNA
WT
tdp-1 (ok803)
J2 IP RNA
WT
tdp-1 (ok803)
Y53C10a.6
Y53c10a.5
nud-2
apb-3
569 bp AS annotated
863 bp AS annotated
B
Antisense transcripts
Direction of transcription
Input RNA
Wild type
tdp-1 (ok803)
J2 IP RNA
Wild type
tdp-1 (ok803)
his-50
his-49
his-51
his-52
his-54
his-53

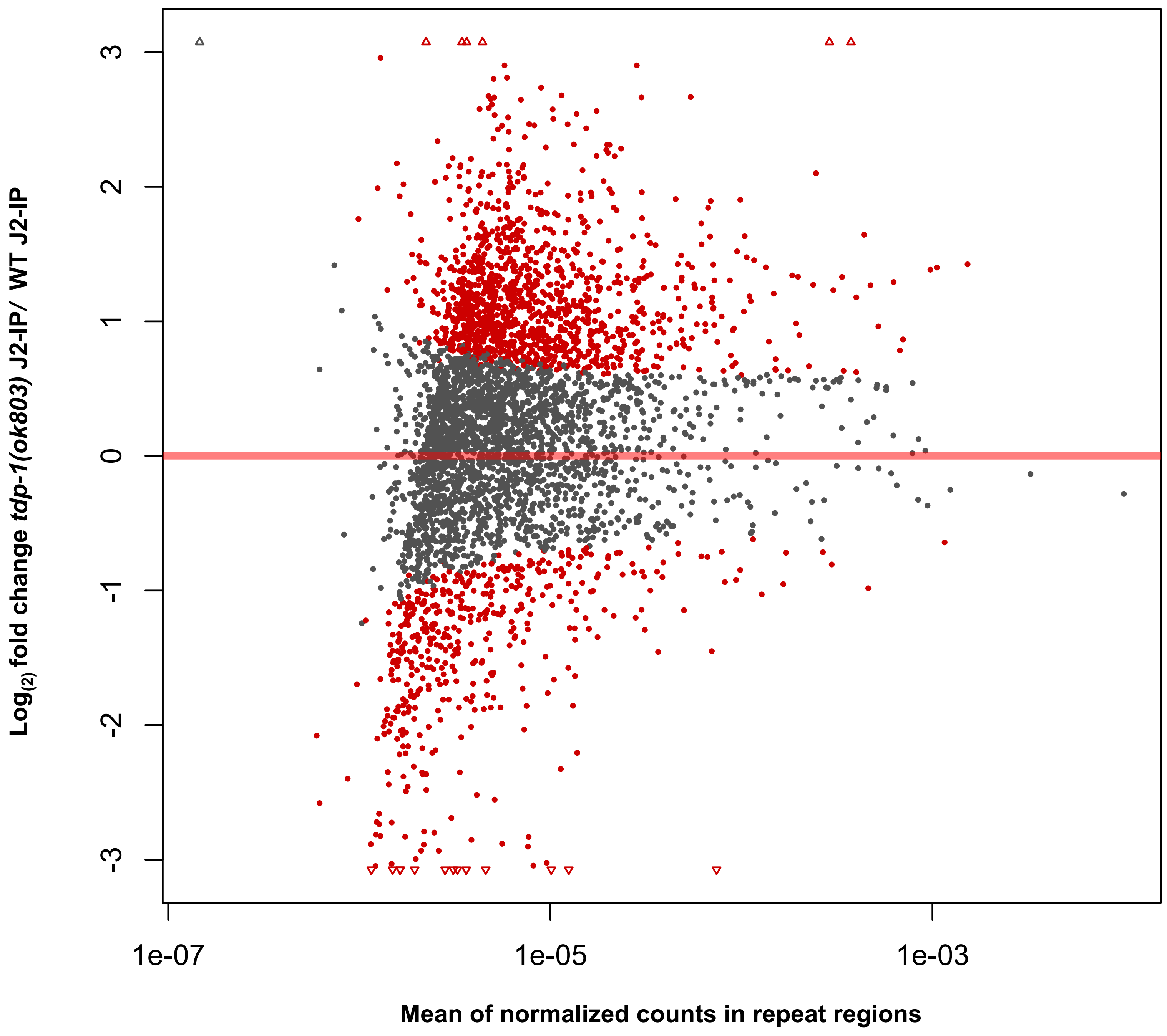

Log(2) fold change tdp-1(ok803) J2-IP/ WT J2-IP
Mean of normalized counts in repeat regions
3
2
1
0
-1
-2
-3
1e-07
1e-05
1e-03

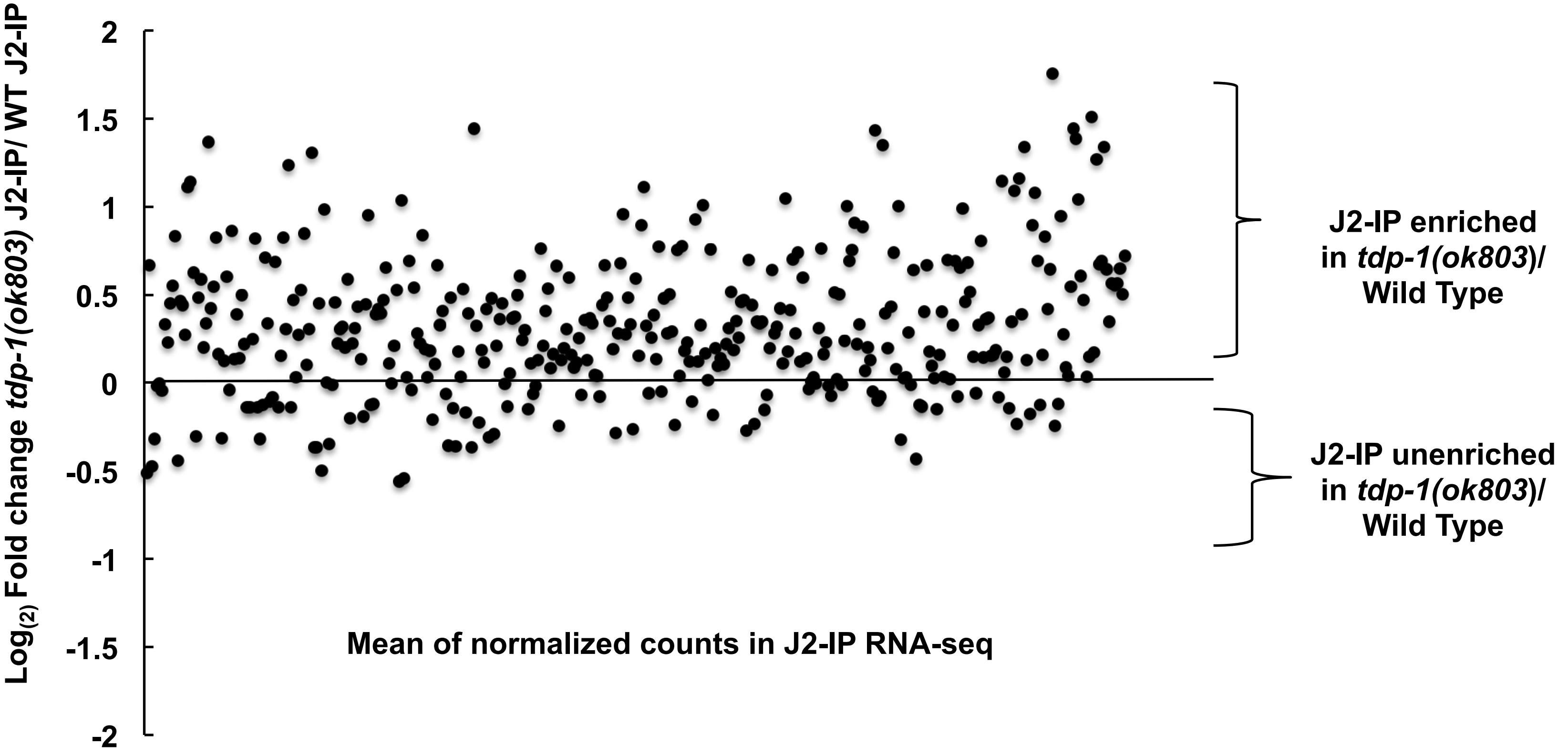
Log(2) Fold change tdp-1(ok803) J2-IP/ WT J2-IP
2
1.5
1
0.5
0
-0.5
-1
-1.5
-2
Mean of normalized counts in J2-IP RNA-seq
J2-IP enriched
in tdp-1(ok803)/
Wild Type
J2-IP unenriched
in tdp-1(ok803)/
Wild Type

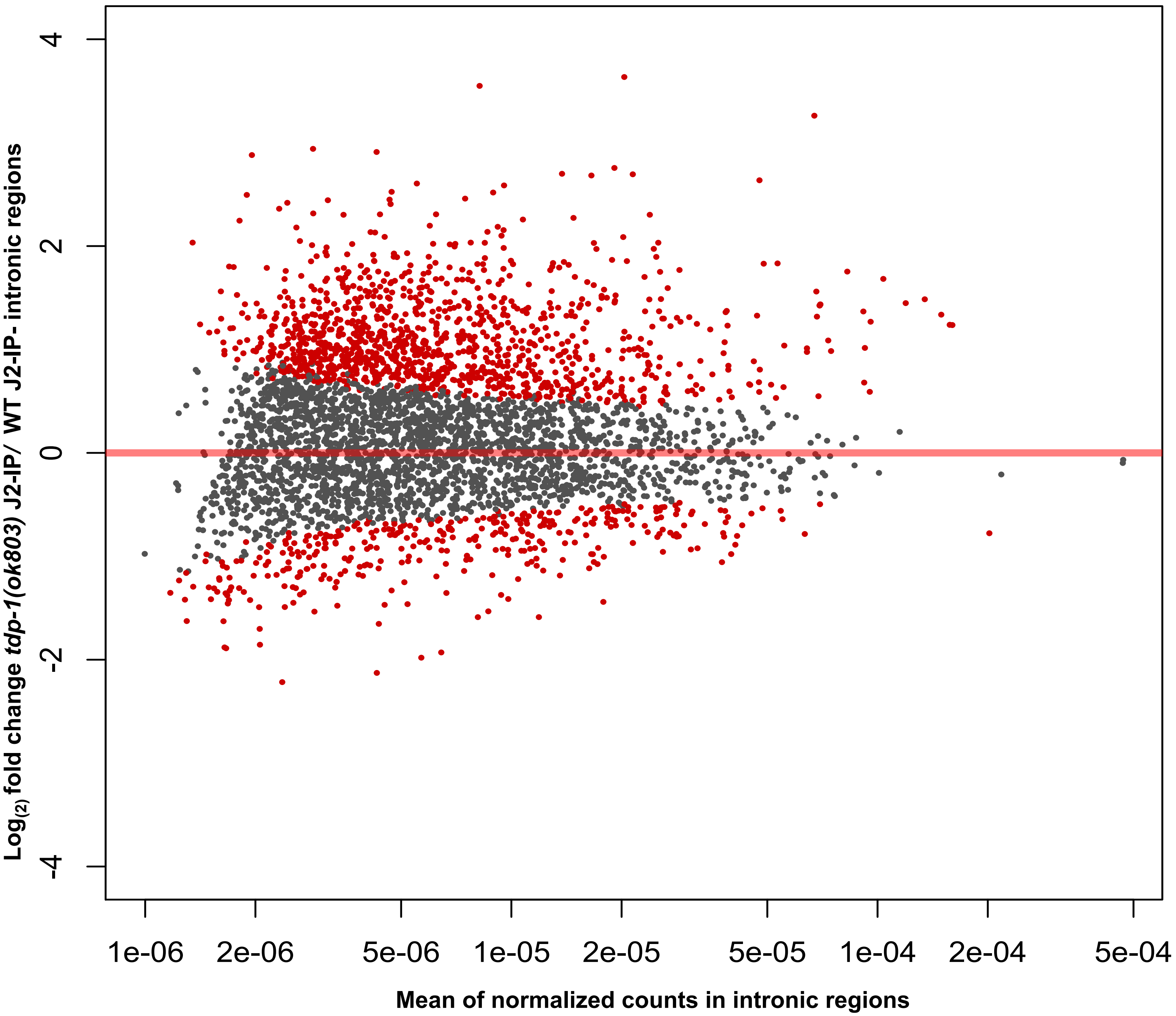

Log(2) fold change tdp-1(ok803) J2-IP/ WT J2-IP- intronic regions
4
2
0
-2
-4
1e-06
2e-06
5e-06
1e-05
2e-05
5e-05
1e-04
2e-04
5e-04
Mean of normalized counts in intronic regions

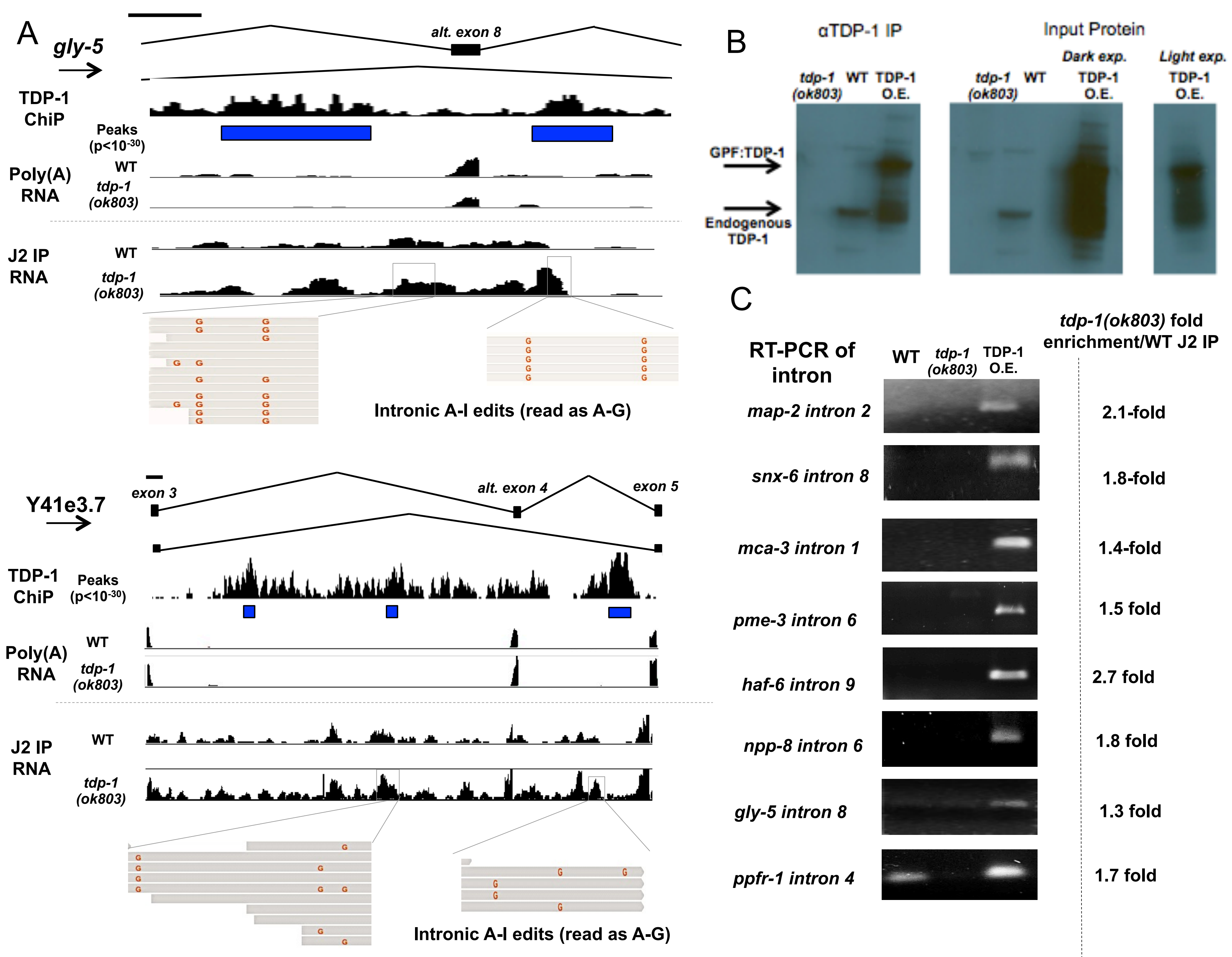
A
gly-5
alt. exon 8
TDP-1 ChiP
Peaks (p<10-30)
Poly(A) RNA
WT
tdp-1 (ok803)
J2 IP RNA
WT
tdp-1 (ok803)
Intronic A-I edits (read as A-G)
Y41e3.7
exon 3
alt. exon 4
exon 5
TDP-1 ChiP
Peaks (p<10-30)
Poly(A) RNA
WT
tdp-1 (ok803)
J2 IP RNA
WT
tdp-1 (ok803)
Intronic A-I edits (read as A-G)
B
αTDP-1 IP
Input Protein
Dark exp.
Light exp.
tdp-1 (ok803)
WT
TDP-1 O.E.
GPF:TDP-1
Endogenous TDP-1
C
RT-PCR of intron
WT
tdp-1 (ok803)
TDP-1 O.E.
tdp-1(ok803) fold enrichment/WT J2 IP
map-2 intron 2
2.1-fold
snx-6 intron 8
1.8-fold
mca-3 intron 1
1.4-fold
pme-3 intron 6
1.5 fold
haf-6 intron 9
2.7 fold
npp-8 intron 6
1.8 fold
gly-5 intron 8
1.3 fold
ppfr-1 intron 4
1.7 fold

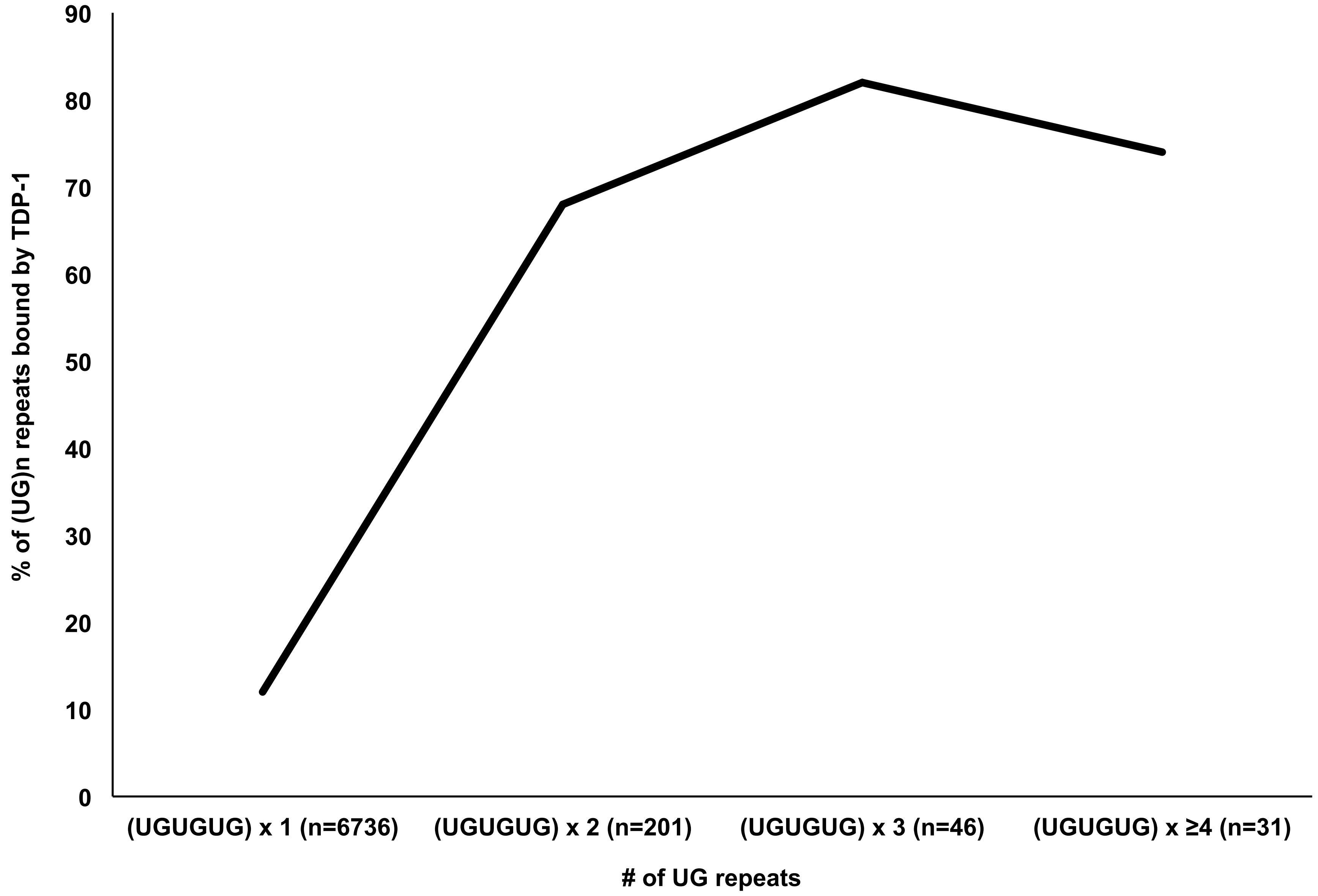

90
80
70
60
50
40
30
20
10
0
% of (UG)n repeats bound by TDP-1
(UGUGUG) x 1 (n=6736)
(UGUGUG) x 2 (n=201)
(UGUGUG) x 3 (n=46)
(UGUGUG) x ≥4 (n=31)
# of UG repeats

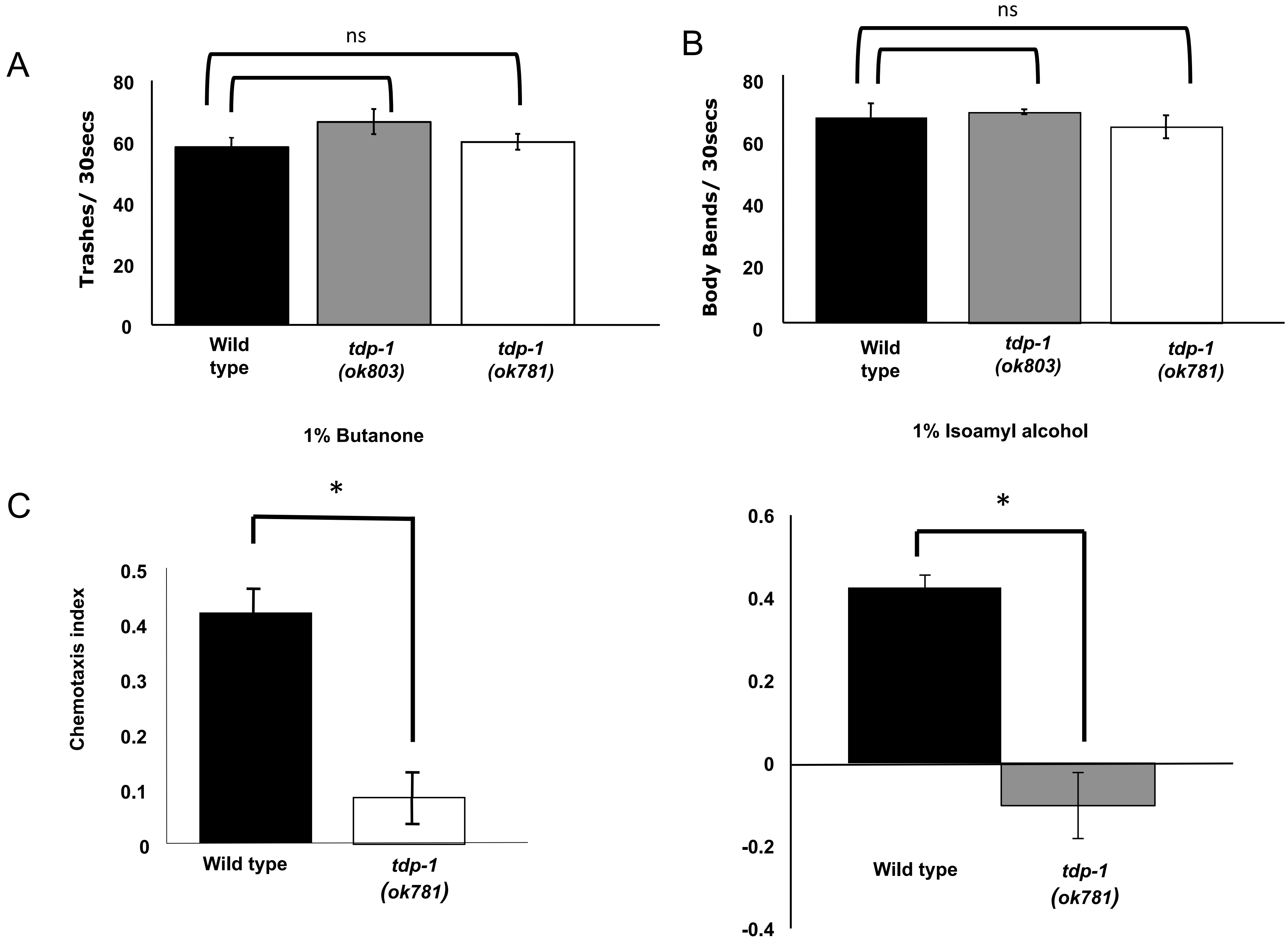
A
ns
80
60
40
20
0
Trashes/ 30secs
Wild type
tdp-1 (ok803)
tdp-1 (ok781)
1% Butanone
B
ns
80
60
40
20
0
Body Bends/ 30secs
Wild type
tdp-1 (ok803)
tdp-1 (ok781)
1% Isoamyl alcohol
C
*
0.5
0.4
0.3
0.2
0.1
0
Chemotaxis index
Wild type
tdp-1 (ok781)
*
0.6
0.4
0.2
0
-0.2
-0.4
Wild type
tdp-1 (ok781)

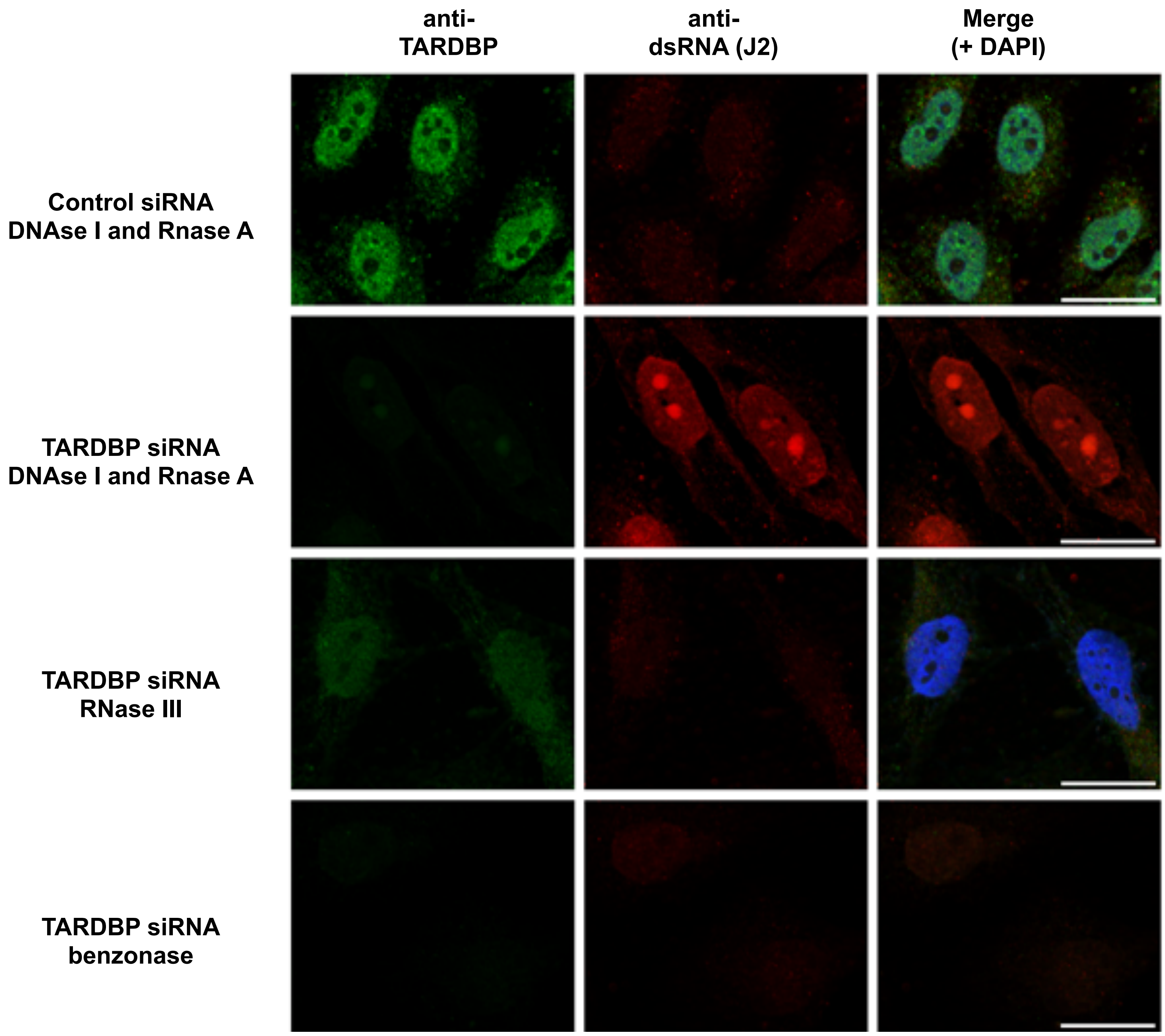
anti-
TARDBP
anti-
dsRNA (J2)
Merge
(+ DAPI)
Control siRNA
DNAse I and Rnase A
TARDBP siRNA
DNAse I and Rnase A
TARDBP siRNA
RNase III
TARDBP siRNA
benzonase

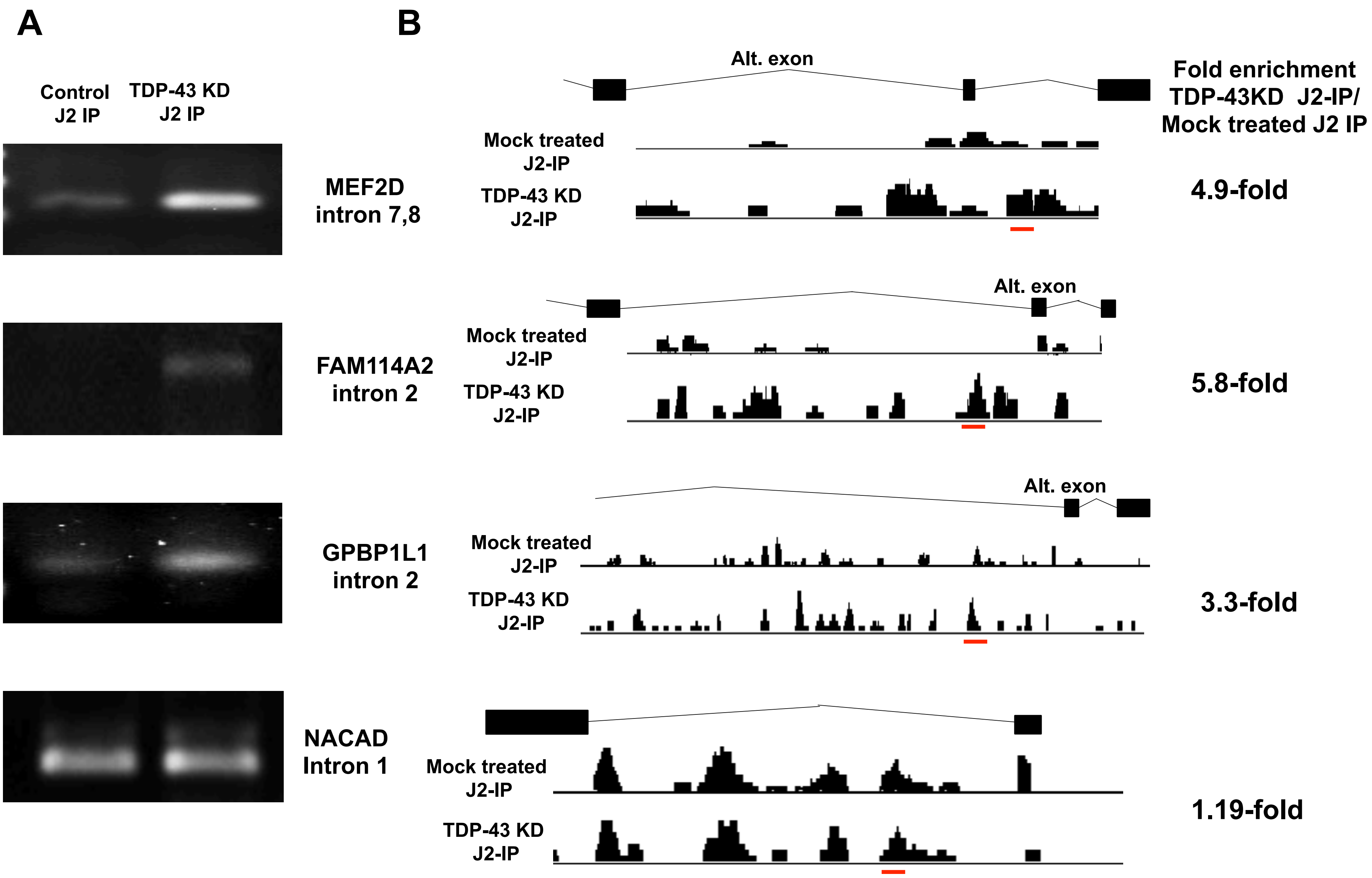
A
B
Control
J2 IP
TDP-43 KD
J2 IP
MEF2D
intron 7,8
FAM114A2
intron 2
GPBP1L1
intron 2
NACAD
Intron 1
Alt. exon
Fold enrichment
TDP-43KD J2-IP/
Mock treated J2 IP
Mock treated
J2-IP
TDP-43 KD
J2-IP
4.9-fold
Alt. exon
Mock treated
J2-IP
TDP-43 KD
J2-IP
5.8-fold
Alt. exon
Mock treated
J2-IP
TDP-43 KD
J2-IP
3.3-fold
Mock treated
J2-IP
TDP-43 KD
J2-IP
1.19-fold

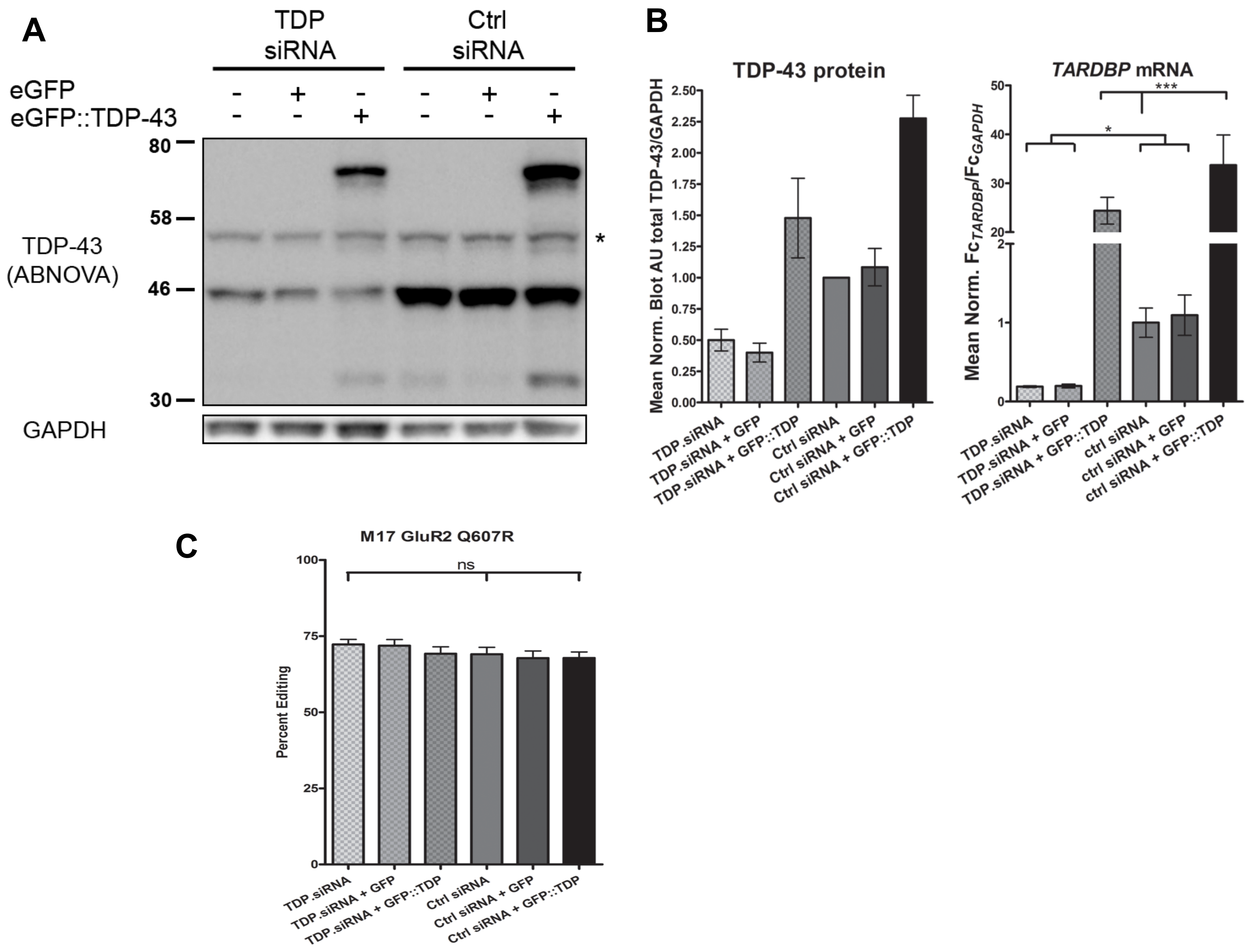

A
TDP siRNA
Ctrl siRNA
eGFP
eGFP::TDP-43
80
58
46
30
TDP-43 (ABNOVA)
GAPDH
*
B
TDP-43 protein
Mean Norm. Blot AU total TDP-43/GAPDH
TDP.siRNA
TDP.siRNA + GFP
TDP.siRNA + GFP::TDP
Ctrl siRNA
Ctrl siRNA + GFP
Ctrl siRNA + GFP::TDP
TARDBP mRNA
Mean Norm. Fc TARDBP/Fc GAPDH
ctrl siRNA
ctrl siRNA + GFP
ctrl siRNA + GFP::TDP
*
***
C
M17 GluR2 Q607R
Percent Editing
ns
