# Supplementary material for: TDP-1, the Caenorhabditis elegans ortholog of TDP-43, limits the accumulation of double-stranded RNA
Source: EMBO J. 2014 Nov 12;33(24):2947–66. doi: 10.15252/embj.201488740 (PMC4282642; doi:10.15252/embj.201488740)
Supplement: Supplementary file 13 — Supplementary Table S12 [file embj0033-2947-sd13.pdf]

Table S12:

Strains used or created during the course of this study are listed below:

Strain	Genotype	Source
**N2- CGCB**	*Wild type*	CGC
**CL6311**	*tdp-1(ok803) II X8 outcross to CGC- N2B*	Original allele from CGC; University of Minnesota, MN, outcrossed in house
**XQ8**	*tdp-1(ok781) II X5 outcross*	Parker Lab
**CL2768**	dvIs100 (*hsp-16.2*/GFP::TDP-1 + pRF4 [*rol-6(su1006)]*) (Integrated array: Chromosome unknown)	This study
**CL6172**	*Ptdp-1::GFP, tdp-1::mCherry*	This study
**CL1787**	*rpl-28/NLS:GFP:ADR-2 dsRNA binding domain*	This study
