# Supplementary material for: TDP-1, the Caenorhabditis elegans ortholog of TDP-43, limits the accumulation of double-stranded RNA
Source: EMBO J. 2014 Nov 12;33(24):2947–66. doi: 10.15252/embj.201488740 (PMC4282642; doi:10.15252/embj.201488740)
Supplement: Supplementary file 14 — Supplementary Table S13 [file embj0033-2947-sd14.pdf]

Table S13:

Primer sequences used to follow deletion mutations or for RT-PCRs are listed in table below.

Gene target	Experiment	Sequence
gsa-1	Confirm anti-dsRNA IP RNA-seq enrichment	Fwd: ggggtgcgtcggcgctggcgc<br>Rev: cagccagcggttgttccagatg
unc-61	Confirm anti-dsRNA IP RNA-seq enrichment	Fwd: cggtgaccttcgtgttcatatcggag<br>Rev: gtgggagaagctctgcttgagcgg
rgef-1	Confirm anti-dsRNA IP RNA-seq enrichment	Fwd: gtactgaatattacatcatcgtcatcag<br>Rev: ctccgagagctccatctagag
**TC1**	Confirm anti-dsRNA IP RNA-seq enrichment	Fwd: aaccgttaagcatggaggtg<br>Rev: cacatgacgacgttgaaacc
gfi-1	Confirm anti-dsRNA IP RNA-seq enrichment	Fwd: gtacctccttgaacttggccaaac<br>Rev: gaaacagttgatggctgtgtgg
haf-6	Confirm anti-dsRNA IP RNA-seq enrichment and anti-TDP-1 IP	Fwd: caggctcgtcgtcctcag<br>Rev: ggcggttttcacccaatttc
let-2	Confirm anti-dsRNA IP RNA-seq enrichement	Fwd: gaaatcccataaccaagacctcg<br>Rev: gagagccagtaagacttgtcg
mca-3	anti-TDP-1 IP	Fwd: cgataattggaaacccgtttacgc<br>Rev: agactcgtatacccttaatctgacacc
**ppfr-1**	anti-TDP-1 IP	Fwd: gcgtgtgccccagcagggc<br>Rev: cgctcaacggcgaggaaaggcgcc
snx-6	anti-TDP-1 IP	Fwd: agcctcaaatcgatgaatagttcac<br>Rev: cgcgagagaaattccactttccag
pme-3	anti-TDP-1 IP	Fwd: aggacatttcccatcagaaaggtcttc<br>Rev: cgaaaattcacaagccttgcgtcag
npp-8	anti-TDP-1 IP	Fwd: gagaacatcggcgaacaggcgtgg<br>Rev: agcgcggtcgcccgaccgctc
map-2	anti-TDP-1 IP	Fwd: gagccccgaggagtttggcg<br>Rev: cgaggcaccacggattcaagatc
gly-5	anti-TDP-1 IP	Fwd: gcgattcgctccgcccactc<br>Rev: agcgtcctcgcgctccgcccacttc
pqn-41	Detection of alternative splicing	Fwd: cgtcgcaacataaacaatcgat<br>Rev1: gcaacacgtcgcgttgcttc<br>Rev2: ctgcaactgctgctgttgctgc
tdp-1	Detection of *ok803*	Fwd: ttgcaagcatcgtttccgggtgc

	deletion	Rev: gaattctgtgaacacgacgccatac
tdp-1	Detection of wild type DNA	Fwd: gtgttctctgaggttcgtgag<br>Rev: cagttagcgatcctttgctg
tdp-1	RT-PCR of *tdp-1* deletion alleles (N-term)	Fwd: atggccgacgaaacgccgaag<br>Rev: gacaacgctctgactctctcgctc
tdp-1	RT-PCR of *tdp-1* deletion alleles (C-term)	Fwd: atggccgacgaaacgccgaag<br>Rev: ccactgatgacattctgac
**HervK***	RT-PCR of J2-IP in M17 human cell lysate	Fwd: tccccttggaatactcctgttttYgt<br>Rev: cattccttgtggtaaaactttccaYtg
FAM114A2	RT-PCR of J2-IP in M17 human cell lysate	Fwd: cctcaagcaatcttcccctgcc<br>Rev: gaggaaagagatggtacagtca
GPBP1L1	RT-PCR of J2-IP in M17 human cell lysate	Fwd: cccattctttactcctcttctatcc<br>Rev: gctcacctctgaataaattcac
MEF2D	RT-PCR of J2-IP in M17 human cell lysate	Fwd: ggtgtgtctgaatgaacataatacc<br>Rev: gctgtgcttaggtgccaagt
NCAD1	RT-PCR of J2-IP in M17 human cell lysate	Fwd: gcctcgtccacctcttcctcctcc<br>Rev: ggcgaggccaagattgaggacctg
FUS/TLS	RT-PCR of J2-IP in M17 human cell lysate	Fwd: cccaactcccagcaatgctt<br>Rev: accctaccctttctggaggt
HP1BP3	RT-PCR of J2-IP in M17 human cell lysate	Fwd: ccacatatacaatggtggtccc<br>Rev: ctgggactacaggcgtgcacc
dnj-5	Verification of alternative splicing change in *tdp-1(ok803)* mutants	Fwd(1): gggctacttgcaagaagaacaag<br>Rev(1): gggaactgaactgggagatc<br>Rev(2): cgcacgttggctctcttgtta
ikke-1	Verification of alternative splicing change in *tdp-1(ok803)* mutants	Fwd: cgcaggttgtaagtgcggaatc<br>Rev: gtctagaaaatcagtgtcttctgcc
kin-25	Verification of alternative splicing change in *tdp-1(ok803)* mutants	Fwd(1): gcctgtggcaggttcttttcatcc<br>Rev(1): gcgatggagcagcaggttggtgt<br>Fwd(2): gcctgtggcaggcaatgacagcg<br>Rev(2): cgaatggcggtcttgactggt
pmt-1	Verification of alternative splicing change in *tdp-1(ok803)* mutants	Fwd(1): ggatcaaactgtagccatggtg<br>Fwd(2): tttacaaaaagccatggtgaacg<br>Rev: cggtggtgaatcgcccgattcc
Y69H2.3	Verification of alternative splicing change in *tdp-*	Fwd(1): gtccggctactggctccacac<br>Rev(1): gtcagtcttgccagtcggtg

	1(ok803) mutants	Fwd(2): cagcgacaagagaccaagaatgc<br>Rev(2): ccaccaccagccaccaacctca
gly-5	Verification of alternative splicing change in *tdp-1(ok803)* mutants	Fwd: taatcaattgggtgattcggagac<br>Rev(1): cgacagcatccagtactgatttcc<br>Rev(2): cgcactgttgcgcacctctcctt
Y41E3.7	Verification of alternative splicing change in *tdp-1(ok803)* mutants	Fwd(1): gaaggctataaaagacgcggaac<br>Rev(1): gcagggttggaaggcaaatcc<br>Fwd(2): gacggtgacggtccgggtccc<br>Rev(2): cggatcaacacgtcttgtctgc
col-54	Verification of abundance change in *tdp-1(ok803)* mutant poly(A)-seq	Fwd: tccaaataactgccctccgggag<br>Rev: gcttctcatcttcaaataccaacg
gst-24	Verification of abundance change in *tdp-1(ok803)* mutant poly(A)-seq	Fwd: acgtcgagtttgaggatgttc<br>Rev: ttggcaaggtagcggagaattgca
oac-29	Verification of abundance change in *tdp-1(ok803)* mutant poly(A)-seq	Fwd: cagaccaaactgatagactattgaatgc<br>Rev: aagtgtaagtgagatccatcg
ZC416.2	Verification of abundance change in *tdp-1(ok803)* mutant poly(A)-seq	Fwd: caacccaaatcagccacaag<br>Rev: ctatcacagcacacctctcc
pgp-8	Verification of abundance change in *tdp-1(ok803)* mutant poly(A)-seq	Fwd: tttgtgtttcagttgccgc<br>Rev: agcacttgtcgcttcatctag
W02D7.5	Verification of abundance change in *tdp-1(ok803)* mutant poly(A)-seq	Fwd: tctcgcaaatcagatagttctcc<br>Rev: cgaagtacacgattgcctttg
sip-1	Verification of abundance change in *tdp-1(ok803)* mutant poly(A)-seq	Fwd: aatcttgagggacacgttctc<br>Rev: gatgacggtgtggatgtgat
W02D7.11	Verification of abundance change in *tdp-1(ok803)* mutant poly(A)-seq	Fwd: cacaactccaaggaagacctc<br>Rev: ctgaactcctgtggtctcttg
dod-3	Verification of abundance change in *tdp-1(ok803)* mutant poly(A)-seq	Fwd: agccatgttcccgaatgag<br>Rev: cgatgaattgtttcgaatcctgg

* HervK primer sequences taken from Douville et. al, 2011.
