# Supplementary material for: TDP-1, the Caenorhabditis elegans ortholog of TDP-43, limits the accumulation of double-stranded RNA
Source: EMBO J. 2014 Nov 12;33(24):2947–66. doi: 10.15252/embj.201488740 (PMC4282642; doi:10.15252/embj.201488740)
Supplement: Supplementary file 15 — Supplementary Table S14 [file embj0033-2947-sd15.pdf]

Table S14:

Table lists all sequencing libraries created in this study along with relevant details and mapping parameters.

RNA sample	Library purpose	Primers	Prep kit	Run type	Mapping parameters
**N2-CGC B L4-20C (x2)**	Gene abundance quantification	Poly(A)	Illumina true seq.	100-single	Tophat2 --b2-very-sensitive -i 30 -I 5000 --read-edit-dist 3 -N 3 --read-realign-edit-dist 0 --segment-length 25 --segment-mismatches 2 --no-coverage-search --min-coverage-intron 30 --max-coverage-intron 5000 --min-segment-intron 30 --max-segment-intron 5000
***tdp-1(ok803)* L4 20C (x2)**	Gene abundance quantification	Poly(A)	Illumina true seq.	100-single	Tophat2 --b2-very-sensitive -i 30 -I 5000 --read-edit-dist 3 -N 3 --read-realign-edit-dist 0 --segment-length 25 --segment-mismatches 2 --no-coverage-search --min-coverage-intron 30 --max-coverage-intron 5000 --min-segment-intron 30 --max-segment-intron 5000
**N2-CGC B L4-20C**	Alternative splicing analysis	Poly(A)	Illumina true seq.	76-Paired-high depth	Tophat2 --b2-very-sensitive -r 198 --mate-std-dev 18 -i 30 -I 5000 --read-edit-dist 3 -N 3 --read-realign-edit-dist 0 --segment-length 25 --segment-mismatches 2 --no-coverage-search --min-coverage-intron 30 --max-coverage-intron 5000 --min-segment-intron 30 --max-segment-intron 5000
***tdp-1(ok803)* L4-20C**	Alternative Splicing Analysis	Poly(A)	Illumina true seq.	76-Paired-high depth	Tophat2 --b2-very-sensitive -r 195 --mate-std-dev 18 -i 30 -I 5000 --read-edit-dist 3 -N 3 --read-realign-edit-dist 0 --segment-length 25 --segment-mismatches 2

					--no-coverage-search --min-coverage-intron 30 --max-coverage-intron 5000 --min-segment-intron 30 --max-segment-intron 5000
**N2-CGC B L4-20C (x3)**	A-to-I RNA editing analysis	random primed	Illumina true seq.	100-Paired	Bfast – See **Supplemental Table 16**
***tdp-1(ok803)* L4 20C (x3)**	A-to-I RNA editing analysis	random primed	Illumina true seq.	100-Paired	Bfast – See **Supplemental Table 16**
***adr-2(gv42)* L4 20C**	A-to-I RNA editing analysis	random primed	Illumina true seq.	100-Paired	Bfast – See **Supplemental Table 16**
**N2-CGC Young Adult-15C input RNA and J2-IP'ed RNA x3)**	J2-IP enrichment identification	random primed	scriptseq v2 strand specific	100-single	*Tophat --b2-very-sensitive -i 30 -I 5000 --read-edit-dist 3 -N 3 --read-realign-edit-dist 0 --segment-length 25 --segment-mismatches 2 --no-coverage-search --min-coverage-intron 30 --max-coverage-intron 5000 --min-segment-intron 30 --max-segment-intron 5000.*
***tdp-1(ok803)* Young adult (Input RNA and J2-IP'ed RNA x3)**	J2-IP enrichment identification	random primed	scriptseq v2 strand specific	100-single	*Tophat --b2-very-sensitive -i 30 -I 5000 --read-edit-dist 3 -N 3 --read-realign-edit-dist 0 --segment-length 25 --segment-mismatches 2 --no-coverage-search --min-coverage-intron 30 --max-coverage-intron 5000 --min-segment-intron 30 --max-segment-intron 5000.*
**N2- CGC B Young Adult-20C (x2 and X1 with RNase)**	Identification of TDP-1 sites of co-transcriptional association	-	ChIP-Seq DNA Sample Prep Kit (IP-102-1001)	50-single	bowtie -l 47 -n 1 -y --best

**Human M17 cells mock treated or treated with Tardbp siRNAs**	J2-IP enrichment identification	random primed	Scriptseq v2 strand specific (RT 55-59C)	100-Paired	Trim 1 off 5', 10 off 3', filter 20 tophat2 --b2-very-sensitive -N 2 --read-realign-edit-dist 0 --library-type frsecondstrand -p 10 -r 157 --mate-std-dev 50 --segmentlength 25 --segmentmismatches 2 --nocoverage-search
