# Supplementary material for: TDP-1, the Caenorhabditis elegans ortholog of TDP-43, limits the accumulation of double-stranded RNA
Source: EMBO J. 2014 Nov 12;33(24):2947–66. doi: 10.15252/embj.201488740 (PMC4282642; doi:10.15252/embj.201488740)
Supplement: Supplementary file 16 — Supplementary Table S15 [file embj0033-2947-sd16.pdf]

Table S15:

Table list *Bfast* parameters used in mapping of total RNA to capture RNA editing and allow downstream quantification.

Input Parameter	Value
FASTQ file	48: Input_file.fq
Conditional (refGenomeSource)	1
Select a reference from history	25: ws220_genomic.fa
Specify the mask	111111111111111111
Hash Width	14
Specify the mask	1111101110111010100101011
Hash Width	14
Specify the mask	10111101011010010110000110100011
Hash Width	14
Specify the mask	10111001101001100100111101010001
Hash Width	14
Specify the mask	111110110111011110111
Hash Width	14
Specify the mask	111111100101001000101111101
Hash Width	14
Specify the mask	111101011100101000101011010101
Hash Width	14
Specify the mask	111101101011011001100000101101
Hash Width	14
Specify the mask	11110110100010001101011001011001
Hash Width	14
Specify the mask	111101001011011010111001011
Hash Width	14
Do not index lower case sequences	False
Conditional (indexing_option)	0
Conditional (params)	1
Truncate key size in 'match'	-1
The maximum number of matches to allow before a key is ignored	75
The maximum number of matches to allow before a read is discarded	400
The strands to consider	Both strands
Scoring Matrix file used to score the alignments	No dataset
Perform ungapped local alignment	False
Perform unconstrained local alignment	False
The number of bases before and after each hit to consider in local alignment	20
The average mismatch quality	10
Conditional (localalign_params)	Unique alignments

Disallow pairing	True
Reverse paired ends	False
Suppress the header in the output SAM file	False
