# Supplementary material for: TDP-1, the Caenorhabditis elegans ortholog of TDP-43, limits the accumulation of double-stranded RNA
Source: EMBO J. 2014 Nov 12;33(24):2947–66. doi: 10.15252/embj.201488740 (PMC4282642; doi:10.15252/embj.201488740)
Supplement: Supplementary file 17 — Supplementary Methods [file embj0033-2947-sd17.pdf]

Supplementary Methods:

Detailed Sequence processing and alignment

Read quality for each sequenced library was ascertained using the FASTQC program from Babraham Bioinformatics (http://www.bioinformatics.babraham.ac.uk/projects/fastqc/). Samples with average Sanger base call quality below 20 at the 5' or 3' ends were trimmed prior to alignment. Reads mapping to genes encoding ribosomal RNAs were removed from all sequence files. See **Table S14** for detailed parameters for each library.

Poly(A) selected library mapping and gene expression analysis

Poly(A) selected libraries were aligned to the WS220 *C. elegans* genome using Tophat v2.0.1(Kim et al, 2013) with the following parameters: *tophat --b2-very-sensitive -i 30 -I 5000 --read-edit-dist 3 -N 3 --read-realign-edit-dist 0 --segment-length 25 --segment-mismatches 2 --no-coverage-search --min-coverage-intron 30 --max-coverage-intron 5000 --min-segment-intron 30 --max-segment-intron 5000*. Multi-mapped reads were allowed for each sample using Tophat's *-g 20* default parameter. Raw read counts for alignment files were obtained using HTseq-count (version 0.5.4p3)(Simon Anders, 2014) with the mode set to intersection-nonempty for each independent replicate. An in-house WS220 GFF file was used for annotation. Differential expression calls were made across 2 independent biological replicates each of wild type and *tdp-1(ok803)* mutant using the Bioconductor package edgeR (release 2.12). For edgeR, we used a pairwise comparison and estimated dispersion with the tagwise dispersion function. An exact test for the negative binomial distribution was used to calculate *p-values*, and a Benjamin-Hochberg adjusted *p-value* was used to identify all significant ($p< 0.05$) genes.

Total RNA mapping

To capture A-to-I RNA editing in total RNA-seq reads, we used the Blat-like Fast Accurate Search Tool (BFAST) (Homer et al, 2009) to align wild type, *tdp-1(ok803)*, and *adr-2(gv42)* total RNA sequences to WS220 *C. elegans* genome (BFAST parameters in **Table S15)**.

J2-IP and Input RNA-seq mapping and gene expression analysis

J2-IP and Input libraries were prepared in triplicate from young adult stage, whole animal lysate either immunoprecipitated with the J2 antibody or lysate dissolved directly in TRizol reagent. After sequencing, filtering and trimming of reads, sequences were aligned to the WS220 *C. elegans* genome using Tophat v2.0.10 with the following parameters: *tophat --b2-very-sensitive -i 30 -I 5000 --read-edit-dist 3 -N 3 --read-realign-edit-dist 0 --segment-length 25 --segment-mismatches 2 --no-coverage-search --min-coverage-intron 30 --max-coverage-intron 5000 --min-segment-intron 30 --max-segment-intron 5000*. Multi-mapped reads were allowed for each sample (due to the repetitive nature of most dsRNA sequences) using Tophat's *-g 20* default parameter. To map reads containing splice leader and or adaptor sequences, we realigned the unmapped reads reported fromTophat2 using bowtie v2.1.0 local alignment: *bowtie2 –local --very-sensitive-local*. The Tophat2 and bowtie2 alignments were then merged together using Picards MergeBamAlignment tool. All J2-IPs and inputs were mapped in the identical fashion for both wild type and *tdp-1(ok803)* libraries.

J2 gene enrichment analysis

Gene counts, intron counts and repeat region (transposons, retrotransposons, inverted repeats and tandem repeats) counts for all J2-IP and input datatsets were generated by intersecting the SAM alignment files with a WS220 gff annotation file containing the chromosome, start coordinate, and end coordinates for each annotated region (extracted from *wormbase*). Genes with a read depth of less than 10 across the entire transcript were filtered out. Introns and repeat regions with less than 20 reads were also filtered out. All genes, introns, and repeat regions passing the filtering criteria were converted to RPKM values prior to statistical analysis.

J2-IP enrichment in wild type compared to input RNA

For J2-IP enrichment in wild type samples, a *two-tailed Student's T-test*, corrected for FDR using the *Benjamin-Hochberg* method across RPKMs of wild type inputs and J2-IPs, was used to determine significant changes between input and J2-IP samples. Genes and repeat regions with >1.5-fold increase in J2-IP RNA-seq compared to input RNA-seq ($p<0.05$, $FDR <0.1$) were considered enriched by J2-IP. *DESeq* algorithm was not appropriate to analyze wild type J2-IP samples against wild type inputs as fold changes in the J2-IP-RNA-seq/ input-RNA-seq did not follow a normal distribution (i.e.

most genes were under represented and most repeat regions were over represented in J2-IP RNA-seq).

J2-IP enrichment in tdp-1(ok803) compared to wild type

The generation of read counts and filtering criteria for genes, introns, and repeat regions was performed in the same manner as the preceding paragraph. To analyze differential J2-IP enrichment in *tdp-1(ok803)* mutant compared to wild type, first average fold changes between wild type and *tdp-1(ok803)* inputs were calculated. The fold change ratio was used to correct read counts in J2-IP RNA-seq samples for each gene, intron and repeat region to eliminate differential enrichment calls in J2-IP due only to relative increased or decreased abundance in total RNA. The counts for each gene, intron (only introns >250 base pairs long were considered to eliminate artifacts) and repeat region in the J2-IP samples were then rounded to the nearest whole number for import into *DESeq v1.14.0 (Anders & Huber, 2010)* for differential J2-IP enrichment testing. For intron counts, all reads mapping to either an exon or a non-coding RNA within that intron were removed prior to import into *DEseq.* To normalize the read counts in *DESeq*, we used the number of mapped reads (after ribosomal RNA subtraction) for each sample as the size factor. Dispersion was estimated using method=per-condition, sharingMode=fit-only, and fitType=local. DESeq's standard res=nbinomTest was used for differential expression calls. Genes were considered differentially enriched for $p<0.05$ *and FDR* <0.1. A gene expression cutoff of $1X10^{-5}$ (Mean of normalized counts- see **Supplementary Figure S10**) was used for high confidence differential gene expression changes. Based on the intron and repeat region dispersion plot, no further gene expression cutoffs were applied to these outputs. Regions showing differential representation in J2-IP RNA-seq samples were further filtered to only report genes, introns and repeat regions that were >1.5-fold enriched by J2-IP compared to the input in both or either wild type or *tdp-1(ok803)* J2-IP.

Identification of *adr-2* dependent editing locations

For wild-type, *adr-2(gv42),* and *tdp-1(ok803)* total RNA samples the alignments were piled up using an in house program. We required a minimum base quality of 20, minimum coverage of 10x and a non-reference base proportion between 0.3 and 0.99. Potential edited sites where more than 90% of the reads mapped had the same start and end position were discarded. Sites where more than 90% of the alignments fell within

3bp from the end of the read were also discarded. For each remaining site the strand was inferred to determine the edited base using WS220 gene annotations. Positions where the strand could not be determined or the strand was ambiguous were discarded. Each of the potential edited positions from the wild-type or *tdp-1(ok803)* samples were compared to the *adr-2(gv42)* sample at the same position and the site was identified as a an edit if the alternate base proportion in the *adr-2(gv42)* sample was < 0.05. All regions identified as edited were visually inspected on the *IGV browser* to confirm the validity of each target. Regions containing reads with multiple base changes (>1 in 100bp read) besides A-to-I conversions (seen as A-to-G compared to the genomic sequence for positive genes and T-to-C for negative genes) were not counted as edited. Editing events mapping to spice junctions were also disregarded. To identify editing in introns enriched by J2 immunoprecipitation that were not identified in the original analysis (due to insufficient read depth), reads mapping to all J2-IP enriched introns were inspected visually on the *IGV genome browser* and hand-annotated as edited. Intronic sequences were counted as edited if the reads mapping to that intron contained at least 10 reads that encompassed 3 or more independent editing events (indicative of promiscuous editing of highly dsRNA) and these editing events were represented in all three biological replicates of wild type and/or *tdp-1(ok803)* J2-IP. Again, intronic regions where reads contained multiple changes besides A-to-I conversions compared to the genomic sequence were disregarded as artifact. Identification of inverted repeats/repetitive elements within edited regions was determined by matching an interval file of all edited regions with an interval file containing the coordinates of all repetitive regions in the *C. elegans* genome (taken from *wormbase*, *genome browser, Repeat Masker track*). We required an overlap of at least 50bp between the edited region and the inverted repeat to consider the two regions overlapping.

Calculation of RNA editing frequency in *tdp-1(ok803)* mutants compared to wild type

The Integrative Genome Viewer (IGV) tool, count, was used to transform BFAST alignment files into pileups for downstream analysis. Edit locations (**Table S4**) were used to compare percent editing between wild type and *tdp-1(ok803)*. Only regions containing a minimum of 20 reads/region in both strains were used. The total number of edited nucleotides in each location was divided by the total number of potentially edited nucleotides for each location. A site was considered potentially edited if the site showed

evidence of an A-to-I change (read as A-to-G (positive strand genes) or T-to-C (negative strand genes) compared to genomic sequence) in either wild type or *tdp-1* mutant RNA-seq. Significant changes in editing were determined by performing a *2-proportional Z-Tests* between wild type and *tdp-1(ok803)* percent edited ratios; $p<0.05$ for significant changes.

Supplementary References

Anders S, Huber W (2010) Differential expression analysis for sequence count data. *Genome biology* 11: R106

Homer N, Merriman B, Nelson SF (2009) BFAST: an alignment tool for large scale genome resequencing. *PloS one* 4: e7767

Kim D, Pertea G, Trapnell C, Pimentel H, Kelley R, Salzberg SL (2013) TopHat2: accurate alignment of transcriptomes in the presence of insertions, deletions and gene fusions. *Genome biology* 14: R36

Simon Anders PTP, Wolfgang Huber. (2014) HTSeq — A Python framework to work with high-throughput sequencing data
bioRxiv preprint
