# Supplementary material for: TDP-1, the Caenorhabditis elegans ortholog of TDP-43, limits the accumulation of double-stranded RNA
Source: EMBO J. 2014 Nov 12;33(24):2947–66. doi: 10.15252/embj.201488740 (PMC4282642; doi:10.15252/embj.201488740)
Supplement: Supplementary file 18 — Legends for Supplementary Figures [file embj0033-2947-sd18.pdf]

Supplementary Figure Legends:

Figure S1: Molecular analysis of *tdp-1* deletion alleles.

A. Figure depicts a schematic of the *tdp-1* locus. Exons are represented by green boxes and introns by black lines. The two annotated transcripts originating from the *tdp-1* locus are shown (a. and b. respectively). The location of the RNA recognition motifs within the gene are indicated by purple bars and the location of the two deletion alleles, *ok803* (red) and *ok781* (blue) are shown below. Primer pair locations used in RT-PCR (see **B)** are indicated by small arrows.

B. Agarose gel shows RT-PCR of the *tdp-1* transcript isolated from wild type, *tdp-1(ok781)* and *tdp-1(ok803)* animals. The transcript targeted and primer pair used in PCR amplification for each RT-PCR is shown on the left. Note that RT-PCR directed against the N-terminal transcript b. produces a product in *tdp-1(ok781)* mutant RNA, but not *tdp-1(ok803)* mutant RNA. Primer sequences are in **Supplementary Table S13**.

C. Western blot of wild type, *tdp-1(ok781)* and *tdp-1(ok803)* total protein. The top panel was probed with anti-TDP-1 antibodies and the bottom panel was probed with an anti-tublin antibody (as a loading control).

D. Transgenic animal expressing Green Florescent Protein driven by the *tdp-1* promoter demonstrating ubiquitous expression of TDP-1 in adult animal.

Figure S2: Confirmation of abundance changes in *tdp-1(ok803)* poly(A)-seq compared to wild type poly(A)-seq by qRT-PCR.

Graph represents qRT-PCR data showing the fold change in abundance of *tdp-1(ok803)* over wild type (y-axis) for a select set of genes (x-axis) that were shown to have abundance differences in poly(A) RNA-seq data. qRT-PCR was done on oligo-dT primed cDNA from wild type and *tdp-1(ok803)* mutant RNA in triplicate. Data was normalized using two different control genes (*pmp-3*: black bars and *cdc-42*: red bars) that were also assayed in triplicate. Fold changes were calculated using the delta/delta CT method and error bars= SEM. Primer sequences are in **Supplementary Table S13**.

Figure S3: Confirmation of transcripts with altered splice site representation in *tdp-1* mutants identified by RNA-seq.

Figure shows the results of semi-quantitative RT-PCR on RNA from wild type and *tdp-1(ok803)* animals (left panel) detecting differentially represented splice forms for transcripts (depicted in the right panel) shown to be altered in abundance by poly(A) RNA-seq. RT-PCRs were done in

triplicate (only one replicate shown). A model depicting the gene structure of the alternative splice is shown to the right and dots indicate that the gene continues in that direction. The gene name is shown and the direction of transcription is indicated by the black arrow. The location of the primers used to generate each PCR band is shown by red arrows. Red arrows over brackets indicate primer sequences that span the splice junction. A total of eight genes were assayed of which one gene failed to show a reproducible change in splice site representation between *tdp-1(ok803)* and wild type (data not shown). Primer sequences are in **Supplementary Table S13**.

Figure S4: Anti-dsRNA immunohistochemistry in *tdp-1* mutants.
A. (Left Panels) Single 1uM optical sections of isolated whole-mount intestines probed with J2 anti-dsRNA antibody. Note J2 immunopositive nuclear foci (arrows); size bar = 10 µm. **(Right Panels)** Projection images (10 one µm sections) of anterior region, reflecting global detection of J2 immunopositive foci. This presentation does not allow association of foci with specific nuclei, but highlights the increased detection of dsRNA foci in the *tdp-1(ok803); adr-2(gv42)* mutant; size bar = 20 µm. All images are digital fusions of DIC and digitally deconvolved epifluorescence images. Red = J2 signal, Blue = DAPI nuclear staining.
B. Anti-dsRNA immunostaining (J2) of muscle tissue in wild type, *tdp-1(ok803)* and the non-null allele *tdp-1(ok781).* dsRNA foci (red) are indicated by white arrows and Dapi (blue) shows nuclei. Scale bar = 10 µm.

Figure S5: Figure 3. dsRNA foci are detected by both J2 antibody and and GFP fused to a dsRNA binding domain. Shown are intestinal nuclei of transgenic reporter strain CL1788 [dvEx788(*rpl-28*/NLS::GFP::*adr-2* dsRNA binding domain co-injected with random chromosomal DNA to produce non-tandem array)] fixed and stained with J2 and anti-GFP antibody with or without stress induction of dsRNA foci. Note tight colocalization of J2 staining and the *in vivo* binding of the GFP-tagged dsRNA probe. Size bar = 10 µm.

Figure S6: Representative regions containing high confidence A-to-I RNA editing. Sequencing reads from wild type (top panels) and *adr-2(gv42)* (bottom panels) RNA-seq mapping showing four representative regions scored as positive of A-to-I editing in our analysis. Blue (T-to-C) and orange (A-to-G) lines within sequencing reads (gray) indicate nucleotide differences between the RNA and DNA sequence that are consistent with A-to-I editing and lines of other colors indicate nucleotide changes not consistent with A-to-I editing. Partial gene models with coding regions as black boxes and non-coding regions (5' and 3' UTRs) as black

lines are shown above sequencing reads. The size and type of structural elements contained within displayed regions (taken from *wormbase*) are indicated. The arrow under the gene name/region type (i.e. 3' UTR) indicates the direction of transcription. Scale bar (thin black line) = 250bp.

Figure S7: TDP-1 does not control the transcription or processing of *adr* transcripts
A. Figure shows the coverage tracks (black bumps) from poly(A) RNA-seq for both worm ADAR homologs, *adr-2* (top) and *adr-1* (bottom) in wild type and *tdp-1(ok803)* mutants. The gene models and direction of transcription (black arrows) are shown above the coverage tracks for each gene. Coverage track height was set to the same value relative for both wild type and tdp-1(ok803) and height is proportional to abundance of each region.
B. Western blot of total protein from wild type, *tdp-1(ok803)* and *adr-1(g66)* animals probed for the ADR-1 protein. Blot is also probed for an unrelated protein, CPF-1, as a loading control.

Figure S8: Pairwise comparison of J2-IP RNA-seq replicates. A. Heat map showing the Euclidean distances between J2-IP samples from wild type and *tdp-1(ok803)* mutants as calculated from the variance stabilizing transformation of the count data. Red and orange intersections indicate highly similar samples, while yellow and white intersections indicate dissimilar samples.
B. Principal components analysis. This type of plot is useful for visualizing the overall effect of experimental covariates and batch effects. Our results indicate that no batch effects other than condition (mutant) were found (i.e. *tdp-1(ok803)* J2-IP samples batch together and wild type J2-IP samples batch together).

Figure S9: RT-PCR confirmation of anti-dsRNA enrichment of selected transcripts.
A. Figure shows the results of semi-quantitative RT-PCR on J2 immunoprecipitated RNA from wild type (left panel) and *tdp-1(ok803)* (middle pane) lysate as well as from lysate pretreated with double-stranded RNA specific RNAse V1 (right panel). The gene producing each transcript assayed is indicated on the left and the genotype of the lysate is indicated at the top of each column of images. While only one repeat is shown, enrichment by J2 immunoprecipitation for selected transcripts was confirmed in two biologically independent immunoprecipitations. Primer sequences are in **Supplementary Table S13**.

B. Shows the fold-enrichment by J2 immunoprecipitation over total RNA for each gene in both wild type and *tdp-1(ok803)* mutant RNA-seq. The final gene in the panel (*let-2*) is shown as an

example of a gene showing similar enrichment by J2 immunoprecipitation in both wild type and *tdp-1* mutants to serve as a loading control for the RT-PCR.

Figure S10: Output of *DESeq* algorithm comparing J2-IP enrichment in *tdp-1* mutants vs. wild type. Graph shows the $\log_{(2)}$ fold change of *tdp-1(ok803)* over wild type J2-IP RNA-seq (x-axis) plotted against the mean of normalized (to library size) counts (y-axis). Prior to feeding counts into DESeq, all counts were normalized to input. Each dot represents an individual gene and red dots indicate genes that are significantly (*p<0.05, FDR<0.1*) different in representation between *tdp-1(ok803)* and wild type J2-IP. Triangles indicate genes whose fold change falls outside of the scale of the plot. Data was generated using three biologically independent replicates of each sample.

Figure S11: J2 immunoprecipitation in *tdp-1(ok803)* selects for convergent overlapping transcripts and histone transcripts.

A. Two examples of convergent gene pairs enriched in the *tdp-1(ok803)* J2 immunoprecipitation. Representative sequencing reads mapping to the sense strand (red) antisense strand (blue) are shown. Models indicating the identity of each gene within the convergent pair and the amount of annotated antisense (AS) overlap are below. Reads from input RNA-seq (top panel) and J2 immunoprecipitated RNA-seq (bottom) panel are displayed.

B. Figure shows sequencing reads from input RNA (top panel) and J2 immunoprecipitated RNA (bottom panel) that map to a histone cluster in wild type and *tdp-1(ok803*) mutants. Expression tracks (black bumps) are proportional to the number of reads mapping to the region shown and tracks were set to the same scale for both wild type and *tdp-1(ok803)* mutant RNA-seq samples. Read orientation is indicated as in **A**. Note the antisense transcripts are preferentially recovered in the ok803 J2-IP consistent with the recovery of inter-strand dsRNA. The orientation of each histone gene is shown in the gene model below with colored arrows and reads that map antisense to the annotated gene are indicated by shaded boxes. Scale bar = 250bp for each region shown.

Figure S12: Output of *DESeq* algorithm comparing J2-IP enrichment in *tdp-1* mutants vs. wild type for repetitive regions. Graph shows the $\log_{(2)}$ fold change of *tdp-1(ok803)* over wild type J2-IP RNA-seq (x-axis) plotted against the mean of normalized (to library size) counts (y-axis). Prior to feeding counts into *DESeq*, all counts were normalized to input. Each dot represents an individual repetitive region (taken from *wormbase, genome browser- repeat masker track*) and red dots indicate regions that are significantly (*p<0.05, FDR<0.1*) different in

representation between *tdp-1(ok803)* and wild type J2-IP. Data was generated using three biologically independent replicates of each sample.

Figure S13: Transcripts altered in abundance in *tdp-1(ok803)* poly(A) RNA-seq are enriched for dsRNA structure in *tdp-1* mutants. Graph shows the $\log_2$ fold change of *tdp-1(ok803)* over wild type J2-IP- RNA-seq (y-axis) for all genes significantly altered in representation between wild type and *tdp-1(ok803)* poly(A) RNA-seq (taken from **Supplementary Table S1**). Genes are graphed by increasing average abundance (WT and *tdp-1(ok803)*) in J2-IP RNA-seq (x-axis).

Figure S14: Output of *DESeq* algorithm comparing J2-IP enrichment in *tdp-1* mutants vs. wild type for intronic regions. Graph shows the $\log_{(2)}$ fold change of *tdp-1(ok803)* over wild type J2-IP RNA-seq (x-axis) plotted against the mean of normalized (to library size) counts (y-axis). Prior to feeding counts into *DESeq*, all counts were normalized to input. Each dot represents an individual intronic region and red dots indicate regions that are significantly ($p<0.05$, $FDR<0.1$) different in representation between *tdp-1(ok803)* and wild type J2-IP. Data was generated using three biologically independent replicates of each sample.

Figure S15: TDP-1 associates and limits dsRNA structure/stability of intronic regions
A. Additional examples of genes with excessively structured introns that are misspliced in *tdp-1(ok803)* mutants. The *gly-5* (top) and Y41e3.7 (bottom) transcripts are shown. Expression tracks (black bumps) for TDP-1 ChIP-seq along with blue boxes indicating significant peaks are shown in the top panel for each diagram. Poly(A) RNA-seq (middle panel for each diagram) and J2 immunoprecipitated reads (bottom panel) are also shown. Gene models are shown above and the arrow indicates direction of transcription. Gray boxes indicate areas of A-I RNA editing and representative edited nucleotides (read as A to G) are displayed below. Scale bars are indicated at the top left of each gene model and equal 250bp.
B. Western blot of protein immunoprecipitated by the TDP-1 antibody (left gel) or total protein (right gels) probed for TDP-1. The genotype of the strain assayed is indicated above the gel and arrows indicate either endogenous TDP-1 or the TDP-1:GFP fusion protein (expressed by the TDP-1 over-expression [O.E.] strain). Input protein for the TDP-1 O.E. strain was over loaded and a lighter exposure of this lane is shown to the right.
C. Figure shows RT-PCR directed against J2 enriched intronic regions from RNA transcripts immunoprecipitated with TDP-1 specific antibodies. Anti-TDP-1 immunoprecipitation was done in whole worm extract prepared from *in vivo* UV-cross-linked animals. Parallel

immunoprecipitation was performed on extract from wild type, *tdp-1(ok803)* mutant and TDP-1 over expression animals (see Materials and Methods) and the genotype is indicated above the gel. All samples were extensively treated with DNAse prior to reverse transcription and RT- controls for each PCR showed no signal (data not shown). The intron and gene assayed in each RT-PCR is indicated on the left and the fold-enrichment over wild type for each intronic region in the *tdp-1(ok803)* J2 IP determined by RNA-seq is shown on the right. Primer sequences are in **Supplementary Table S13**.

Figure S16: TDP-1 preferentially associates with UG rich introns. Graph showing the percentage of introns bound by TDP-1 (y-axis) (determined by the presence of a ChIP-seq peak in that intron) that contain increasing numbers of UG repeats (x-axis). *n*= number of expressed introns genome wide containing indicated number of UG repeats.

Figure S17: *tdp-1(ok803)* and the non-null allele *tdp-1(ok781)* have similar phenotypes
A. Bar graph depicting the results of thrashing assays (see Materials and Methods) scored by hand in *tdp-1* mutant worms. The average number of thrashes per 30 seconds (Y-axis) is shown for each genotype indicated (x-axis). An average of 10 adult animals grown at 20C were measured three independent times (blinded X 1). No significant difference between *tdp-1* mutants and wild type was observed.
B. Bar graph showing the result of thrashing assays scored using image J software. Body bends per 30 seconds are graphed on the Y-axis and the genotype tested is indicated on the X-axis. Each assay recorded at least 10 worms and the assay was done three independent times. Again, no significant movement defect was observed in adult animals at 20C.
C. Graphs show the results of chemotaxis assays for both Butanone and Isoamyl alcohol done on the non-null allele, *tdp-1(ok781).* *P<0.001 (Student's T-test); error bars = SEM.

Figure S18: **Specificity of J2 antibody labeling of dsRNA.**

A. In HeLa cells knocked down for TARDBP, the increased dsRNA signal is unaffected by digestion with DNase I (note the absence of DAPI signal) or the single stranded RNA specific RNase A. The dsRNA signal can be selectively deleted with the double stranded RNA specific RNase III or the non-selective nuclease, Benzonase. Exposure times for green and red channels are fixed. Bar = 20μm

Figure S19: Additional examples of increased intronic dsRNA in TDP-43 knockdown cells in transcripts previously shown to have altered splice site representation in ALS pathological tissue.

A. Semi-quantitative RT-PCR of RNA precipitated from J2-IP on lysate from mock treated M17 cells (left panel) and TDP-43 knockdown M17 cells (right panel). RT-PCR was done in triplicate (only one replicate shown). Primer sequences are in **Supplementary Table S13**.

B. Expression tracks from J2-IP RNA-seq in regions shown to have increased recovery in TDP-43 knockdown J2-IP compared to mock treated controls. The gene model for each region is shown above the expression tracks. All expression tracks are normalized to the number of reads in each sample and the height of the track indicates reads depth. The fold enrichment by J2-IP in TDP-43 knockdown over control (normalized to total RNA-seq) for each region is shown to the right. The thin black line indicates scale and scale is set to 500bps. Red lines show the location of primer sequences relative to the gene. NACAD intron one is shown as a region not enriched in TDP-43 knockdown J2-IP over control (as a loading control).

Figure S20: Editing of endogenous GluR2 Q607R is not affected in M17 cell culture by experimental manipulation of TDP-43 levels.

A. Blot of total protein from M17 cells treated with TDP-43 siRNAs or with control siRNAs indicating that TDP-43 knockdown was successful. Recovery of TDP-43 protein levels by expression of eGFP tagged plasmid is also shown. * = non-specific band.

B. Quantification of TDP-43 expression in M17 samples was calculated by densitometry from 3 blots and by qPCR of TARDBP mRNA (N = 4/group). * $P<0.05$, *** $P<0.001$. ANOVA with Bonferroni's post Hoc. Bars = S.E.M. **C.** Percentage editing of hGluR2 Q607R does not correlate with TDP-43 expression. ANOVA with Dunnett's post Hoc test against the control group (Ctrl siRNA) indicates no change in editing due to manipulation of TDP-43 expression. Columns are mean percentage A-to-I editing of GluR2 from 5 samples/treatment group (3 sequence reads/sample) with error bars indicating S.E.M.
