# Supplementary figures and images for: TDP-1, the Caenorhabditis elegans ortholog of TDP-43, limits the accumulation of double-stranded RNA
Source: EMBO J. 2014 Nov 12;33(24):2947–66. doi: 10.15252/embj.201488740 (PMC4282642; doi:10.15252/embj.201488740)

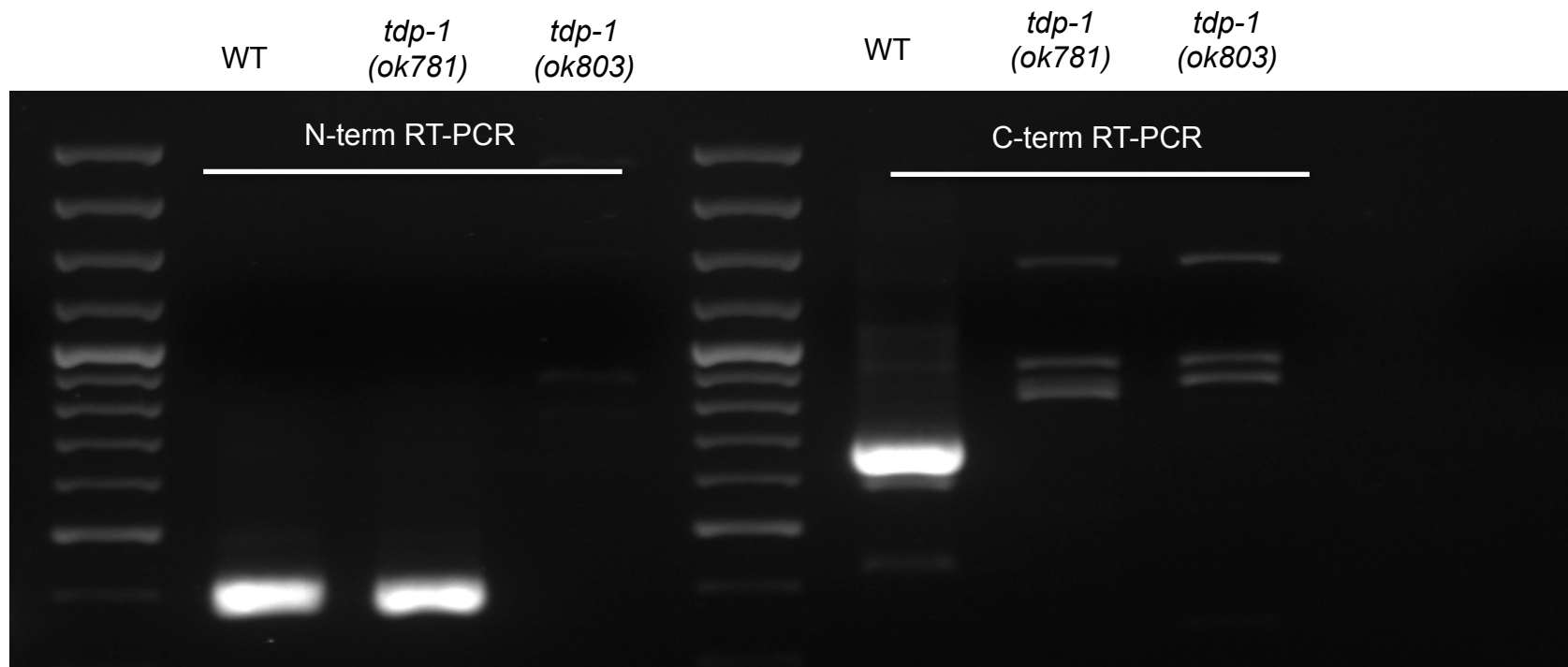

**Anti-TDP-1**

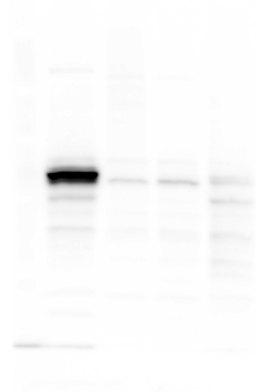

**Anti-Tublin**

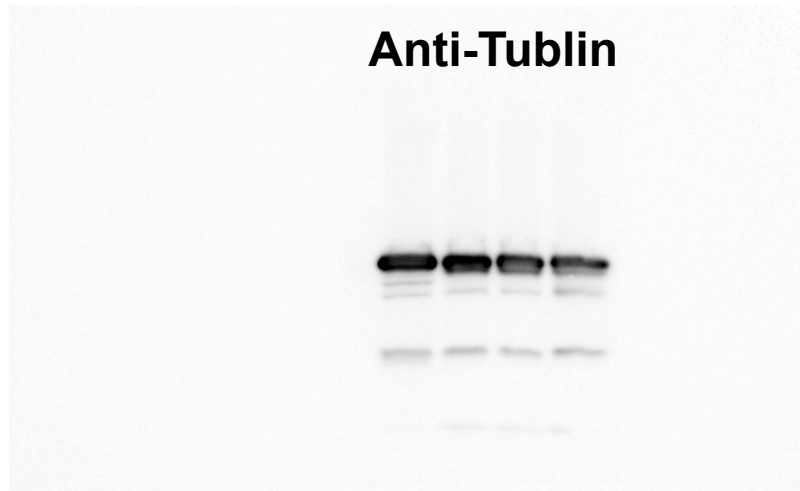

Raw data – Supplemental Figure 1

Supplement: Supplementary file 19 — Source Data for Supplementary Figure S1 [file embj0033-2947-sd19.pdf]

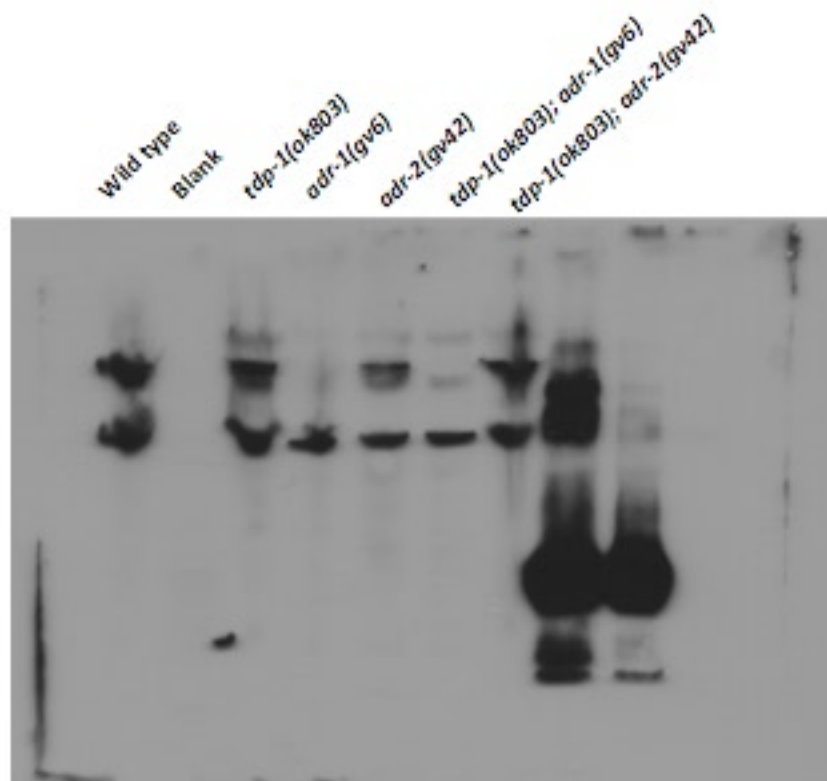

Raw data – Supplemental Figure 7

Supplement: Supplementary file 21 — Source Data for Supplementary Figure S7 [file embj0033-2947-sd21.pdf]

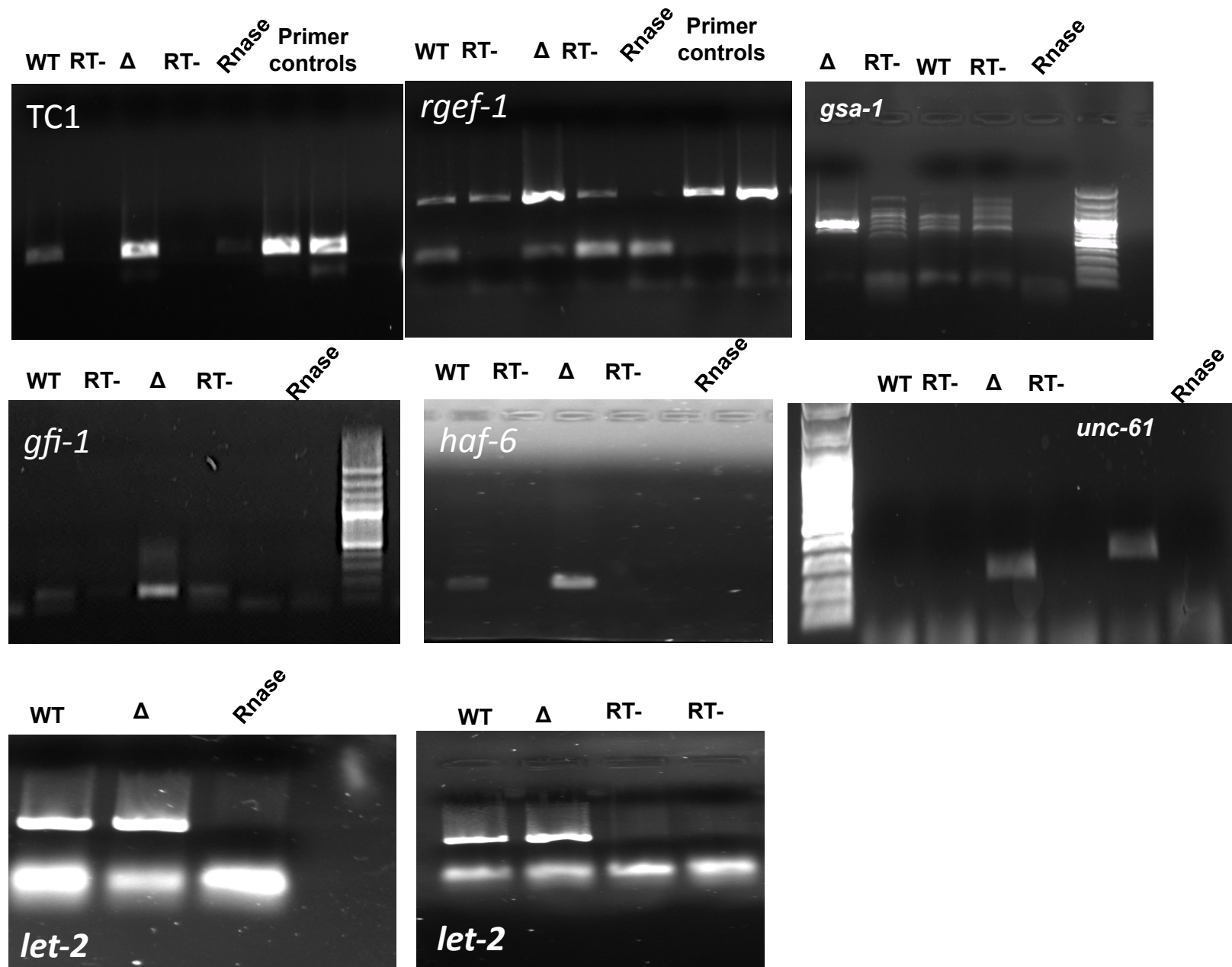

Raw data – Supplemental Figure 9

Supplement: Supplementary file 22 — Source Data for Supplementary Figure S9 [file embj0033-2947-sd22.pdf]

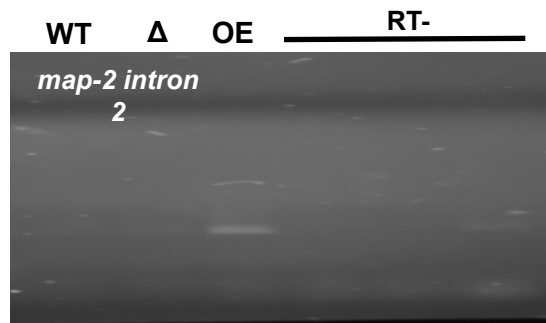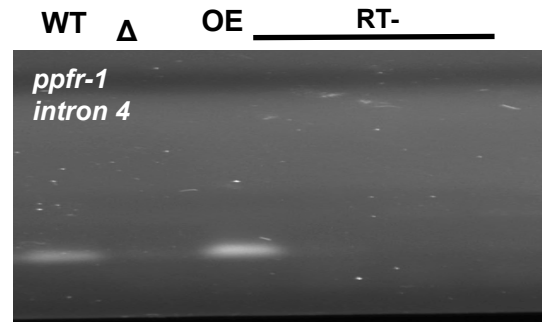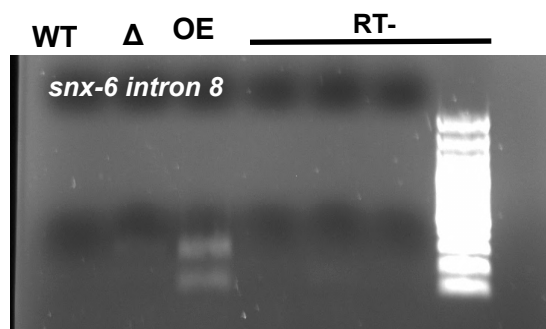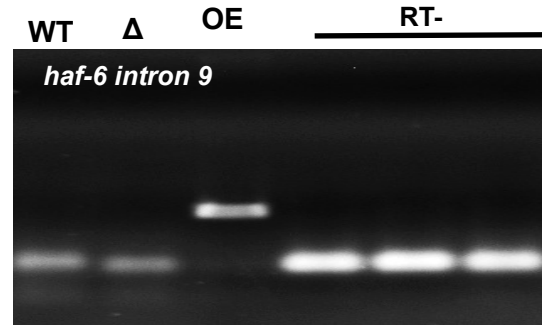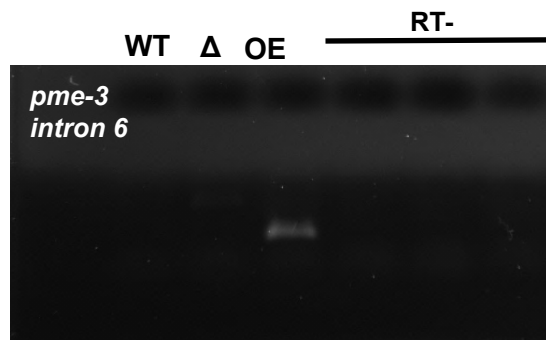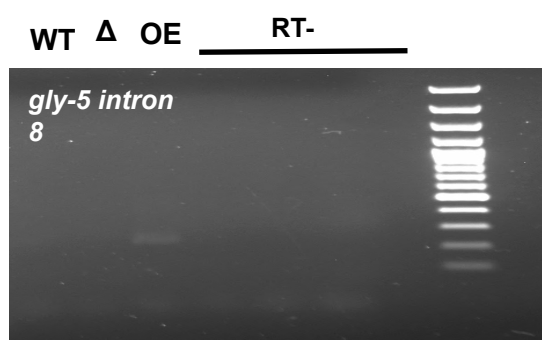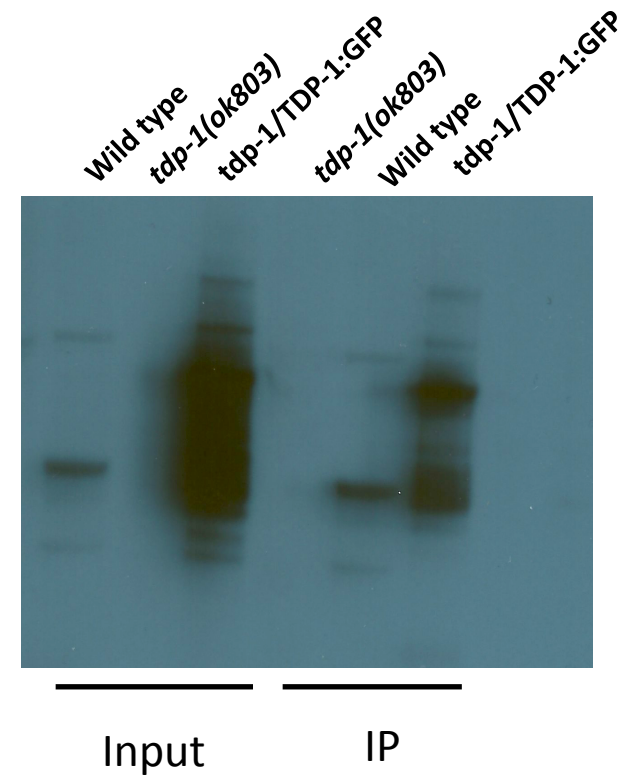

Raw data – Supplemental Figure 15

Supplement: Supplementary file 23 — Source Data for Supplementary Figure S15 [file embj0033-2947-sd23.pdf]

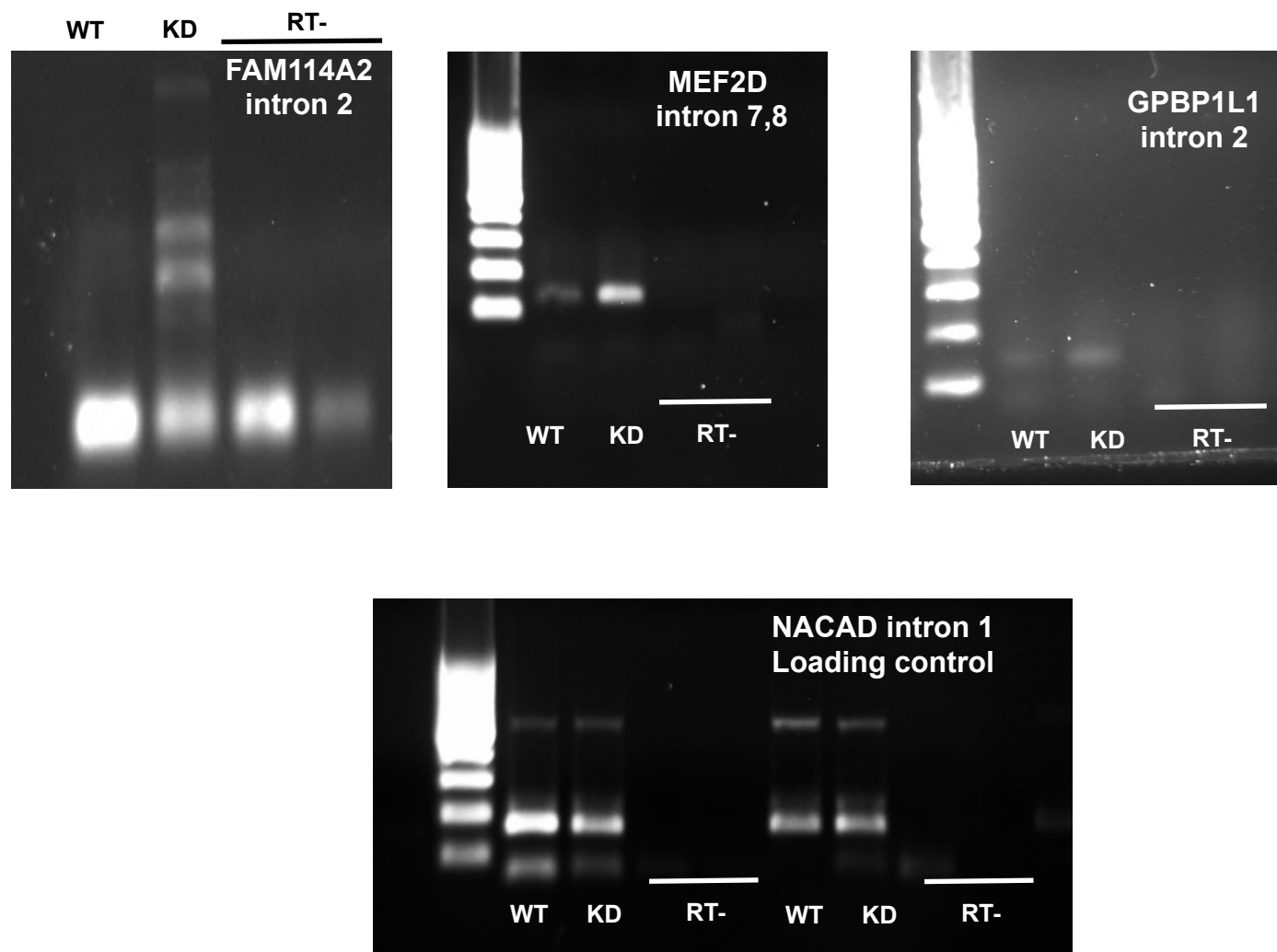

Raw data –Supplemental Figure 19

Supplement: Supplementary file 24 — Source Data for Supplementary Figure S19 [file embj0033-2947-sd24.pdf]

Anti-TDP-43

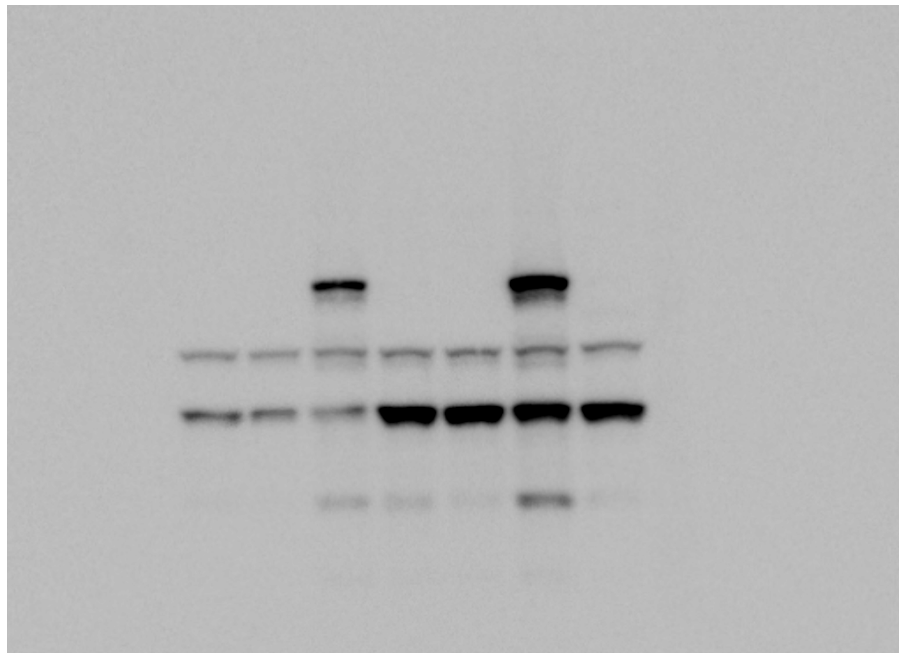

Anti-GAPDH

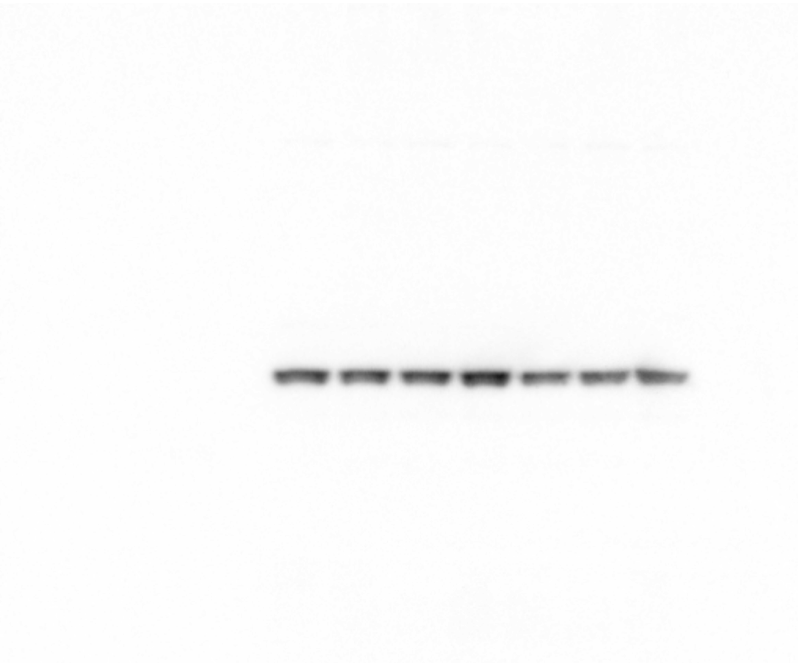

Raw data – Supplemental Figure 20

Supplement: Supplementary file 25 — Source Data for Supplementary Figure S20 [file embj0033-2947-sd25.pdf]

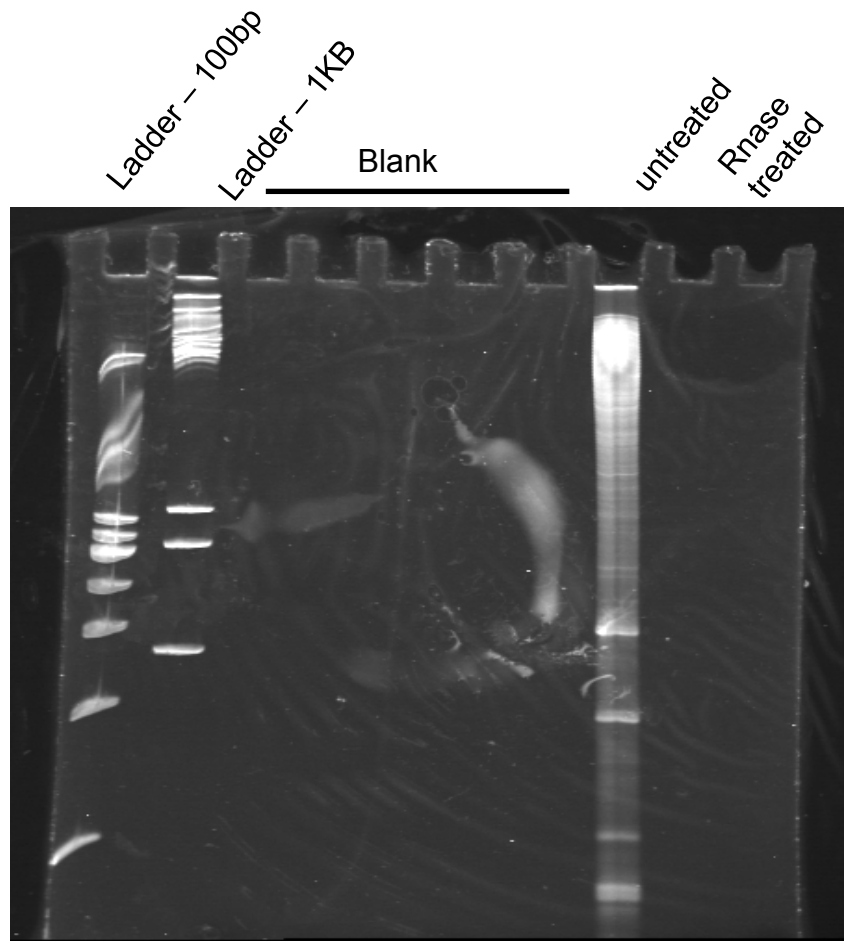

**Confirmation of completion of Rnase treatment in ChIP extract**

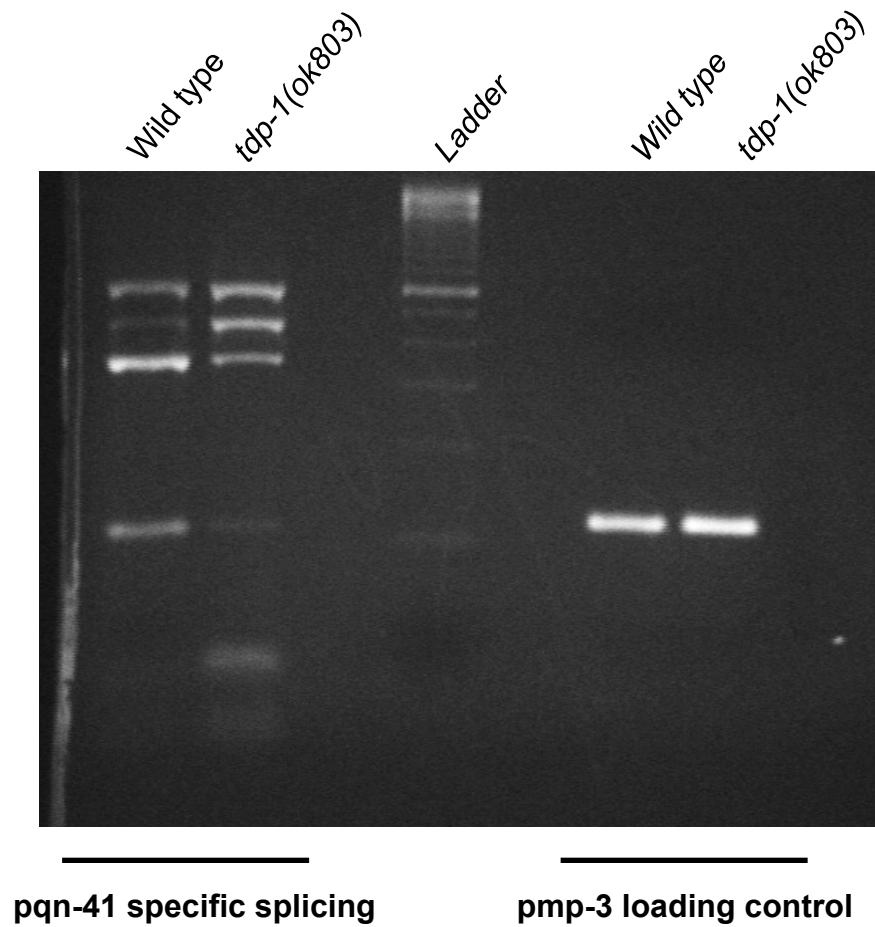

Raw data – Figure 6

Supplement: Supplementary file 27 — Source Data for Figure 6 [file embj0033-2947-sd27.pdf]

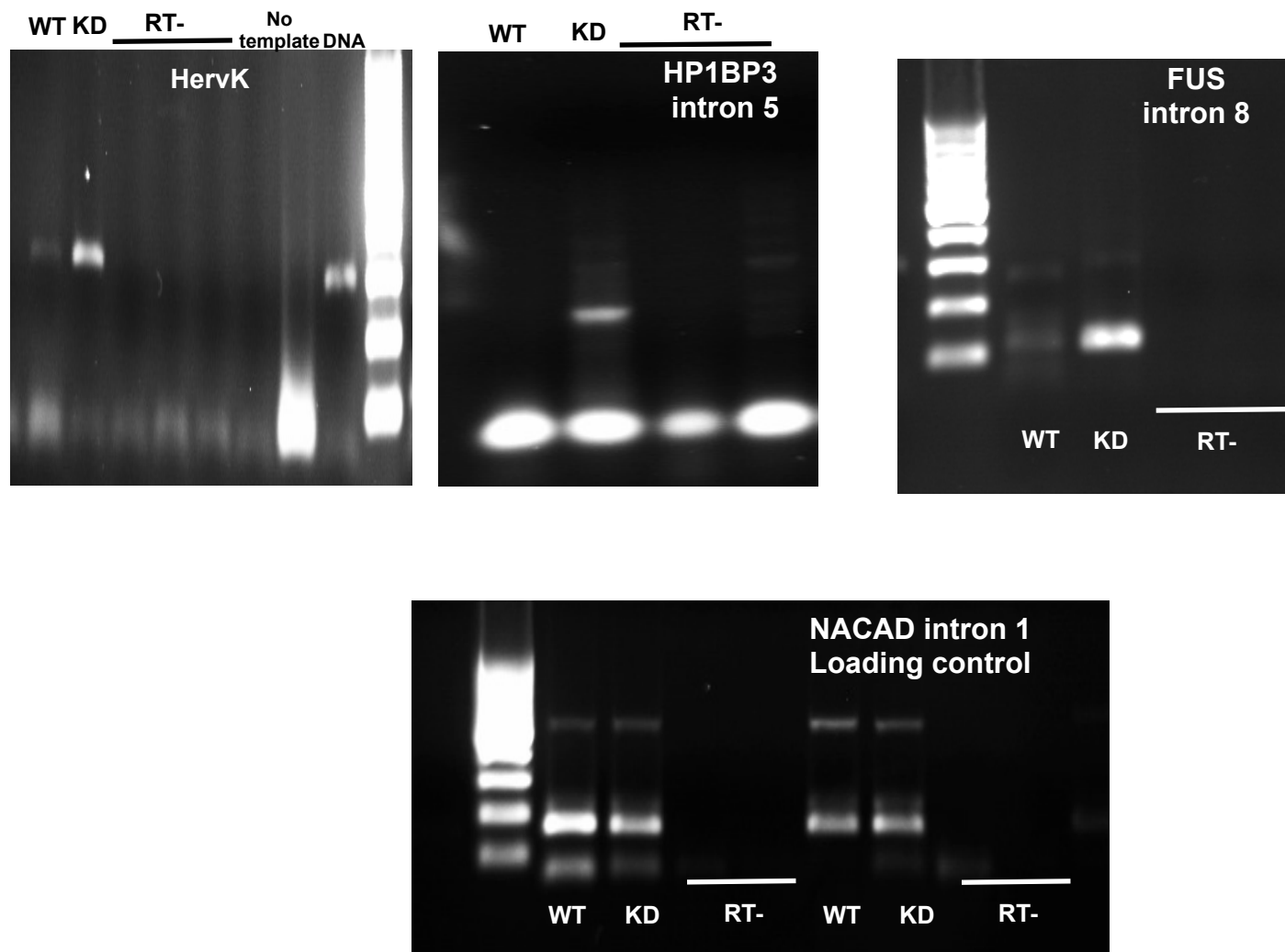

Raw data –Figure 8

Supplement: Supplementary file 28 — Source Data for Figure 8 [file embj0033-2947-sd28.pdf]
