# Supplementary material for: TDP-1, the Caenorhabditis elegans ortholog of TDP-43, limits the accumulation of double-stranded RNA
Source: EMBO J. 2014 Nov 12;33(24):2947–66. doi: 10.15252/embj.201488740 (PMC4282642; doi:10.15252/embj.201488740)
Supplement: Supplementary file 20 — Source Data for Supplementary Figure S3 [file embj0033-2947-sd20.pdf]

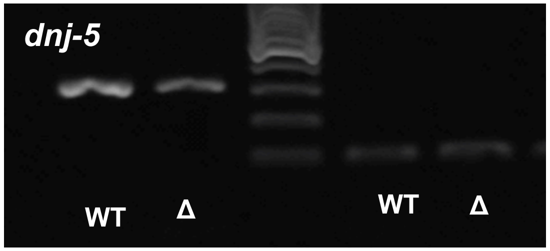

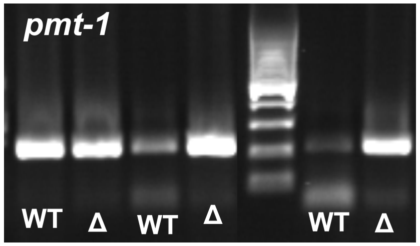

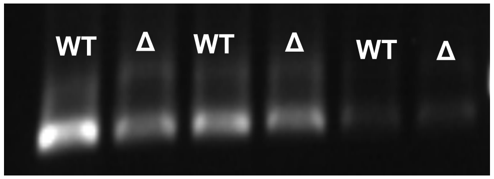

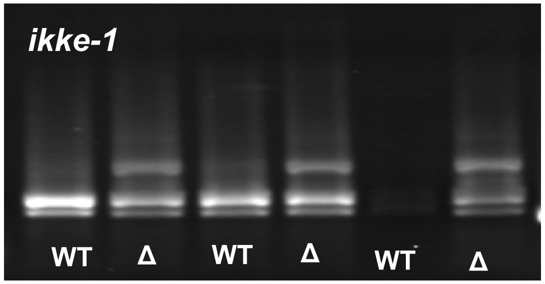

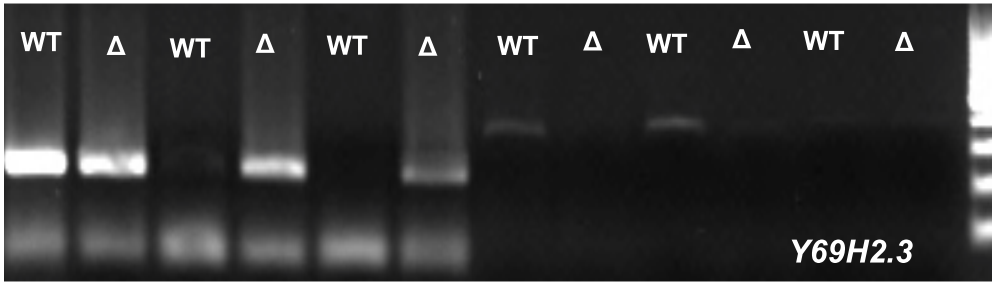

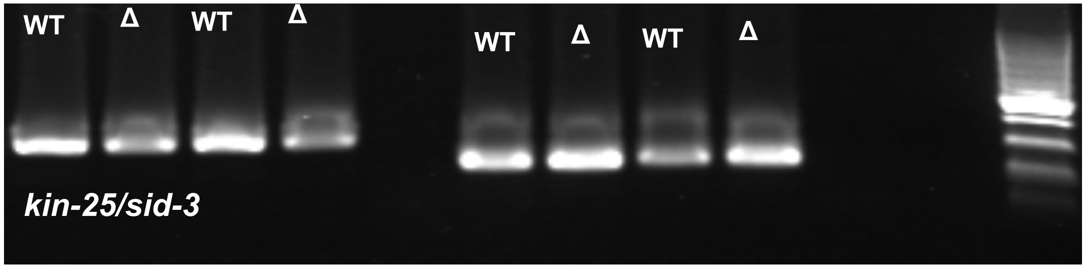

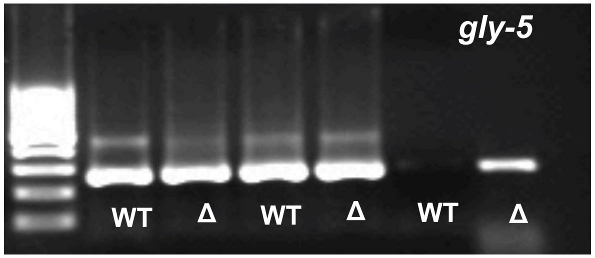

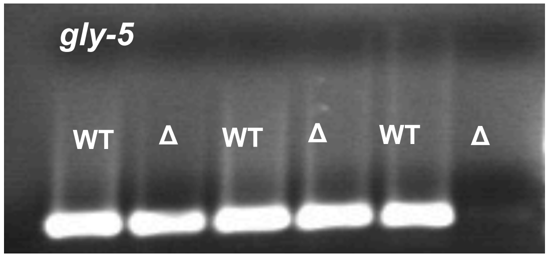

Raw data – Supplemental Figure 3
Reactions were done with decreasing concentrations of cDNA until signal was not blown out (1-3
